# JAB1/CRL4B complex represses PPARG/ACSL5 expression to promote breast tumorigenesis

**DOI:** 10.1038/s41418-025-01642-0

**Published:** 2025-12-12

**Authors:** Ting Hu, Tianyu Ma, Miaomiao Huo, Jiaxiang Liu, Die Zhang, Yu Li, Jinyuan Chang, Min Zhang, Yinuo Wang, Tianyang Gao, Baowen Yuan, Siqi Wang, Qing Li, Xiaoqi Ma, Jingyao Zhang, Wei Huang, Yan Wang

**Affiliations:** 1 State Key Laboratory of Molecular Oncology, National Cancer Center/National Clinical Research Center for Cancer/Cancer Hospital, Chinese Academy of Medical Sciences and Peking Union Medical College, Beijing, China; 2 Department of Biochemistry and Molecular Biology, School of Basic Medical Sciences, Capital Medical University, Beijing, China; 3 Institute of Cancer Research, Henan Academy of Innovations in Medical Sciences, Zhengzhou, Henan China

**Keywords:** Cancer metabolism, Epigenetics

## Abstract

Fatty acid metabolism is critical for tumor progression, supplying bioenergetic and biosynthetic substrates to rapidly proliferating cancer cells. However, the precise mechanisms by which fatty acid metabolism influences breast cancer progression remain unclear. In this study, we aimed to explore the molecular mechanism by which C-Jun activation domain-binding protein-1 (*JAB1*) promotes breast cancer progression through regulating fatty acid metabolism. The JAB1 is identified as an oncogene in breast cancer. JAB1 promotes cell proliferation, invasion, and stemness by stabilizing CUL4B protein. Mechanistically, JAB1 forms a transcriptional repressor complex with the Cullin 4B-Ring E3 ligase (CRL4B) complex, co-occupying the promoters of key fatty acid metabolism genes, *PPARG* and *ACSL5*, thus leading to their transcriptional repression. This activates fatty acid metabolism, increasing mitochondrial oxygen consumption and supporting the energetic demands of tumor cells. Notably, *JAB1* inhibition reverses chemotherapy resistance associated with *CUL4B* overexpression. These findings underscore the pivotal role of JAB1 in regulating breast cancer progression and indicate that JAB1 inhibitors could serve as promising therapeutics for patients with elevated *CUL4B* expression.

## Introduction

Breast cancer remains among the most prevalent malignancies in women globally, with high incidence and mortality rates [1]. Despite significant advances in treatment, tumor metastasis and drug resistance hinder successful outcomes. These phenomena are often associated with genetic mutations, epigenetic alterations, and metabolic disorders [2–4]. Hence, characterizing the mechanisms underlying these processes could provide novel insights and therapeutic strategies.

JAB1 (C-Jun activation domain-binding protein 1), also known as COPS5 (constitutive photomorphogenic homolog subunit 5), is a multifunctional protein that serves as a critical subunit of the COP9 signalosome (CSN). JAB1 overexpression is closely associated with various tumors, promoting tumorigenesis and development by disrupting the cell cycle, DNA repair responses, metabolic regulation, and increasing chemotherapy resistance [5–7]. Additionally, JAB1 helps regulate the ubiquitin–proteasome system, affecting the protein degradation process. Previous studies have shown that JAB1 modulates CRL4 activity by removing NEDD8 modifications and regulating substrate degradation [8, 9].

JAB1 exhibits deubiquitination activity. For instance, JAB1, induced by nuclear factor (NF)-κB p65, is essential for stabilizing PD-L1 mediated by tumor necrosis factor-alpha (TNF-α) in cancer cells [10]. Berberine inhibits the deubiquitinating activity of JAB1, thereby reducing PD-L1 expression in cancer cells and enhancing anti-tumor immunity [11]. Studies have demonstrated that PDIA6 drives pancreatic cancer progression and enables immune evasion through a dual mechanistic role, wherein it destabilizes β-catenin and PD-L1 via its partner JAB1 [12]. Hence, aberrant JAB1 expression may impact the stability of certain tumor-associated proteins, promoting tumor initiation and progression. Increased CUL4B stability is also closely related to tumorigenesis and development. In breast cancer, aryl hydrocarbon receptor (AhR) acts as a ligand-dependent bridge between the stimulator of the interferon gene (STING) and the CUL4B/RBX1 E3 ligase complex, promoting STING degradation and facilitating tumor progression [13]. We previously reported that the CRL4B/NuRD (MTA1) complex is recruited by transcription factors such as SNAIL and ZEB2 to jointly bind to the promoters of E-cadherin and AXIN2, promoting cell invasion and stemness [14]. However, the mechanism regulating CUL4B stability remains unclear.

Fatty acid metabolism provides energy and biosynthetic precursors to tumor cells. Hence, enhanced fatty acid synthesis and oxidation are vital to tumorigenesis, progression, and drug resistance [15, 16]. Tumor cells upregulate the expression of fatty acid synthase (FASN), thereby enhancing fatty acid synthesis, which is utilized to construct cell membranes and organelles and regulate cell growth and survival [17, 18]. Meanwhile, fatty acid oxidation is an important energy pathway for tumor cells, particularly under hypoxic or glucose-deprived conditions; its metabolites also participate in signal transduction and gene expression regulation [19]. Thus, pharmacologically inhibiting the activity or expression of FASN and Acetyl-CoA Carboxylase (ACC) effectively suppresses tumor cell growth [20–22]. Moreover, diets rich in ω-3 polyunsaturated fatty acids can inhibit tumor cell growth and invasion and enhance chemotherapy sensitivity [23–25]. Accordingly, characterizing fatty acid metabolism mechanisms and developing targeted drugs can provide novel tumor treatment strategies.

The present research endeavors to explore the molecular pathways through which JAB1 facilitates the advancement of breast cancer. We found that the JAB1/CRL4B complex transcriptionally represses the expression of fatty acid metabolism-related genes PPARG and ACSL5, thereby promoting the proliferation and invasion of breast cancer cells. Furthermore, CSN5i-3, an inhibitor of JAB1, can effectively reverse the chemotherapy resistance of breast cancer cells induced by CUL4B. In conclusion, these findings suggest that the oncogene JAB1 shows potential as a promising therapeutic target for the treatment of breast cancer.

## Materials and methods

### Cell culture and transfection

The cells were all purchased from the American Type Culture Collection (ATCC, located in Manassas, Virginia, USA) and cultured in Dulbecco’s modified Eagle’s medium (DMEM) containing 10% fetal bovine serum (FBS). The cells were placed in a sterile incubator at 37 °C with 5% carbon dioxide for cultivation. All the cells were transfected using Turbofect (Invitrogen, Carlsbad, CA, USA) for the cell transfection experiments. Cells were transfected with siRNAs using Lipofectamine RNAiMAX Reagent (Invitrogen) according to the manufacturer’s instructions. Every experiment was carried out at least three times in duplicate. The plasmid information is presented in Table S3. The siRNA and shRNA sequences are listed in Table S4.

### Antibodies and reagents

The antibodies utilized in this study encompassed normal rabbit IgG (2729), anti-FLAG (F1408), anti-β-Actin (A1978), anti-CUL4B (C9995), anti-Vimentin (V6630), and anti-Fibronectin (F3648), all sourced from Sigma-Aldrich (St. Louis, MO, USA); anti-JAB1 (sc-13157), anti-DDB1 (sc-25367), anti-PPARG (sc-271392), and anti-ACSL5 (sc-365478) from Santa Cruz Biotechnology (Dallas, TX, USA); anti-*α*-Catenin (610193), anti-*γ*-Catenin (610253), anti-E-cadherin (610181), and anti-N-cadherin (610920) from BD Bioscience (Franklin Lakes, NJ, USA); anti-KLF4 (12173), anti-HA (2367), and anti-Ubiquitin (3936) from Cell Signaling Technology (Danvers, MA, USA); anti-JAB1 (27511-1-AP) was obtained from Proteintech (Rocky Hill, NJ, USA); anti-ROC1 (ab2977), anti-OCT4 (ab19857), anti-c-MYC (ab32072), and anti-NANOG (ab109250) from Abcam (Cambridge, UK); anti-H2AK119ub1 (05-678) from Millipore (Billerica, MA, USA). Dynabeads protein A/G were sourced from Invitrogen/Thermo Fisher Scientific (Waltham, MA, USA), while Glutathione-Sepharose 4B beads were obtained from Gene Pharma (Shanghai, China). The protease inhibitor cocktail came from Roche Applied Science (Penzberg, Germany). Lastly, siRNAs targeting JAB1, PPARG, and ACSL5 were procured from JTSBIO Co., Ltd (Wuhan, China), while short hairpin RNAs (shRNAs) targeting JAB1 and CUL4B were obtained from Shanghai Genechem Co., Ltd (Shanghai, China).

### RNA-sequencing analysis

For 48 h, siRNA was transfected into MDA-MB-231 cells. Following the manufacturer’s instructions, total RNA was extracted using the TRIzol reagent (Roche, Penzberg, Germany). For mRNA library creation and sequencing, the product was shipped to Annoroad Gene Technology (Beijing, China). A quality control phase validated the sequencing data, and R packages such as DESeq2, clusterProfiler, and ggplot2 were used for analysis. DEGs were defined as genes with *p* < 0.05 and fold change ≥ 1.5.

### Real-time quantitative PCR (RT-qPCR) analysis

The RNA-Quick Purification Kit (Esunbio, Shanghai, China) was used to extract total RNA. cDNA was created using the PrimeScriptTM RT Master Mix (TaKaRa Bio, Kusatsu, Shiga, Japan). Relative quantification was performed using the ABI QuantStudio5 instrument. Table S5 lists the primers that were employed for the detection. The raw qPCR data are presented in Supplementary Material: Raw qPCR Data.

### Mouse xenograft models

To create breast cancer xenografts, 5 × 10^6^ cells were injected into the left mammary fat pad of 4-week-old female NOD/SCID mice. The tumor volume was then measured every three days. After 4 weeks, all mice were euthanized in compliance with ethical standards. The animal handling and procedures have been approved by the Experimental Animal Ethics Committee of the Cancer Hospital, Chinese Academy of Medical Sciences (NCC2021A285). Following dissection, the tumor xenografts underwent RT-qPCR analysis, immunohistochemical staining, and measurements of their final volume and wet weight.

### In vivo metastasis models

MDA-MB-231 cells stably expressing firefly luciferase (PerkinElmer, formerly Xenogen Corporation) were infected with lentiviruses carrying either an empty Vector or JAB1 shRNA constructs. A total of 1 × 10⁶ cells were resuspended in PBS and injected into the tail veins of 6-week-old female NOD/SCID mice (5 mice per group). Tumor formation and metastasis were assessed by bioluminescence imaging using the IVIS Imaging System (PerkinElmer) 5 weeks after injection.

### EdU incorporation assays

Ten thousand cells per well were used to seed the treated cells in a 96-well plate. After cell adhesion, the EdU assay was performed on the cells following the guidelines provided by the kit from RiboBio (Guangzhou, China). A fluorescent microscope was used to take pictures of the cells.

### Colony formation assays

Cells were seeded in 6-well plates at 1000 cells/well after treatment. Following 12–14 days of culture, they were fixed with 4% paraformaldehyde for 15 min, stained with 1% crystal violet for 15 min, rinsed, dried, and imaged.

### Transwell assays

Following lentiviral infection, the top chamber wells were seeded with either MDA-MB-231 cells (4 × 10^4^) or MCF-7 cells (2 × 10^5^) in serum-free DMEM media. The chambers were moved to a 24-well plate that contained 10% FBS. The cells on the underside of the membrane were fixed with paraformaldehyde, then stained with crystal violet and counted. For every membrane, three high-power fields were counted.

### Wound-healing assays

Three hundred thousand treated cells were seeded in a six-well plate. Upon reaching 90% confluence, scratches were made with a 200 μL pipette tip; cells were gently rinsed thrice with PBS and photographed under a microscope. After a 24-h incubation in serum-free medium, microscopic images were taken once more.

### Spheroid-forming assays

DMEM-F12 medium without serum, supplemented with 0.4% bovine serum albumin, B27 (50× dilution, Invitrogen), 20 ng/mL basic fibroblast growth factor, 10 ng/mL epidermal growth factor, and 5 μg/mL insulin (Invitrogen) was used to plate 5000 cells per well in six-well ultralow attachment plates. Every 3 days, new aliquots of stem cell media were introduced. After being observed on day 5, mammospheres were left to cultivate until day 15, at which time their size and quantity were assessed under a microscope.

### Immunoprecipitation and western blotting

Collect 5 × 10^7^ cells, lyse them and collect the supernatant. Add normal rabbit/mouse immunoglobulin G (IgG) or specific antibodies to the supernatant and mix for overnight incubation. Subsequently, add Protein A/G Sepharose beads and incubate again. Proteins were then denatured and resolved by 10% SDS-PAGE. The original images of the western blots are presented in Supplementary Material—Uncropped western blots.

### Immunopurification and mass spectrometry

The MDA-MB-231 cells transfected with the JAB1 plasmid carrying a FLAG tag were collected. The cell lysate (5 × 10^8^ cells) was loaded onto a FLAG column and the protein complex was eluted, after which the samples were separated by SDS-PAGE and subjected to silver staining.

### Glutathione S-transferase (GST) pull-down experiments

Glutathione-Sepharose 4B beads were used to create and purify the glutathione S-transferase (GST) fusion protein. An in vitro transcription and translation system utilizing rabbit reticulocyte lysate (TNT system, Promega, USA) was employed to carry out the experiments. In the GST pull-down experiment, a certain volume of GST fusion protein was mixed with the translation products, followed by incubation and washing, and then detected using western blotting.

### Chromatin immunoprecipitation (ChIP) and Re-ChIP

As previously mentioned, ChIP and Re-ChIPs were carried out in MDA-MB-231 cells [26]. In brief, the ChIP experiment involved crosslinking, sonicating, preclearing 5 × 10⁸ cells, and incubating them with the appropriate antibody, followed by DNA extraction and precipitation. After extracting the immune complexes from the beads, they were diluted using the ChIP dilution buffer and subjected to a second immunoprecipitation. Primers for each target gene promoter were used in PCR or qPCR analyses of DNA template enrichment. The sequences of the primers are listed in Table S6.

### Deubiquitination assays

For 36 h, HEK293T cells were transiently transfected using the corresponding plasmids. Denaturing lysis buffer was used to harvest the cells following a 6h incubation period with 10 μM MG132. After diluting the supernatant with washing buffer, it was incubated with anti-FLAG M2 affinity gel on a rotating platform overnight at 4 °C. For immunoblotting analysis, the immunoprecipitants were collected. The cells were exposed to 10 μM MG132 for 8 h before being lysed with RIPA lysis buffer to identify the endogenous ubiquitination of CUL4B. After adding either normal IgG or the primary CUL4B antibody to the supernatant, it was incubated overnight at 4 °C. Subsequently, 25 μL of 50% protein A agarose beads were introduced to the mixture, which was then incubated for a period of six hours. Immunoprecipitation was performed to evaluate the level of CUL4B poly-ubiquitination.

### Drug synergy analysis

The cells, subjected to different treatments, were plated in 96-well plates and exposed to varying concentrations of chemotherapy drugs along with CSN5i-3 (0, 2, 4, 6, and 8 μM) in a matrix layout. After 48 h, the CCK-8 reagent (manufactured by Dojindo) was added, and the absorbance was read. The inhibition rate of the cells was calculated, and according to the ZIP model, the drug synergy score was calculated using SynergyFinder 2.0. The synergistic drug combinations were determined using a web-based tool available at https://synergyfinder.fimm.fi.

### Metabolomics

The sample was removed from −80 °C refrigerator and thawed on ice. The samples were processed by MetWare Company, and the upper layer solution containing free fatty acid methyl esters was collected into injection vials for GC-MS analysis. Metabolomics was performed using the Metware Cloud, a free online platform for data analysis (https://cloud.metware.cn).

### Oxygen consumption rate (OCR), free fatty acid (FFA), and triglyceride (TG) detection

The Seahorse XF Cell Mitochondrial Stress Test Kit (103015-100, Agilent Technologies, Santa Clara, CA, USA) was used to determine the OCR in accordance with the manufacturer’s recommendations. The respective kits were used in accordance with their instructions to assess the concentrations of FFA (S0215S, Beyotime) and TG (S0219S, Beyotime).

### Tissue specimens and immunostaining assays

We purchased tissue samples from Shanghai Outdo Biotech Company (Shanghai, China) and Servicebio Biotechnology Co., Ltd. The study has received approval from the ethical committee (Approval Number: NCC2025C-302), and all participating subjects have signed informed consent forms. After surgical removal, the samples were immediately snap-frozen in liquid nitrogen and kept at −80 °C until they were analyzed. The specimens were first fixed in 4% paraformaldehyde (Sigma-Aldrich) at 4 °C overnight, then embedded in paraffin and cut into 8-micrometer-thick sections. These sections were mounted onto glass slides and subsequently stained for microscopic examination.

### Statistical analysis

The analysis was conducted utilizing GraphPad Prism (version 8.0, GraphPad Software Inc., USA). Unless otherwise specified, the results are presented as the mean ± standard deviation (mean ± SD) from at least three independent experiments. For inter-group comparisons, a two-tailed unpaired Student’s *t* test was employed, while two-way analysis of variance (two-way ANOVA) was used for analyzing differences among three or more groups. The Pearson correlation test was used for correlation analysis. The breast tumor datasets (GSE72653, GSE20713, GSE9893, and GSE65194) were obtained from http://www.ncbi.nlm.nih.gov/geo. Survival data were retrieved from http://kmplot.com/analysis and analyzed using the Kaplan–Meier method followed by the log-rank test.

## Results

### *JAB1* is an oncogene that promotes breast cancer progression

**Fig. 1:**
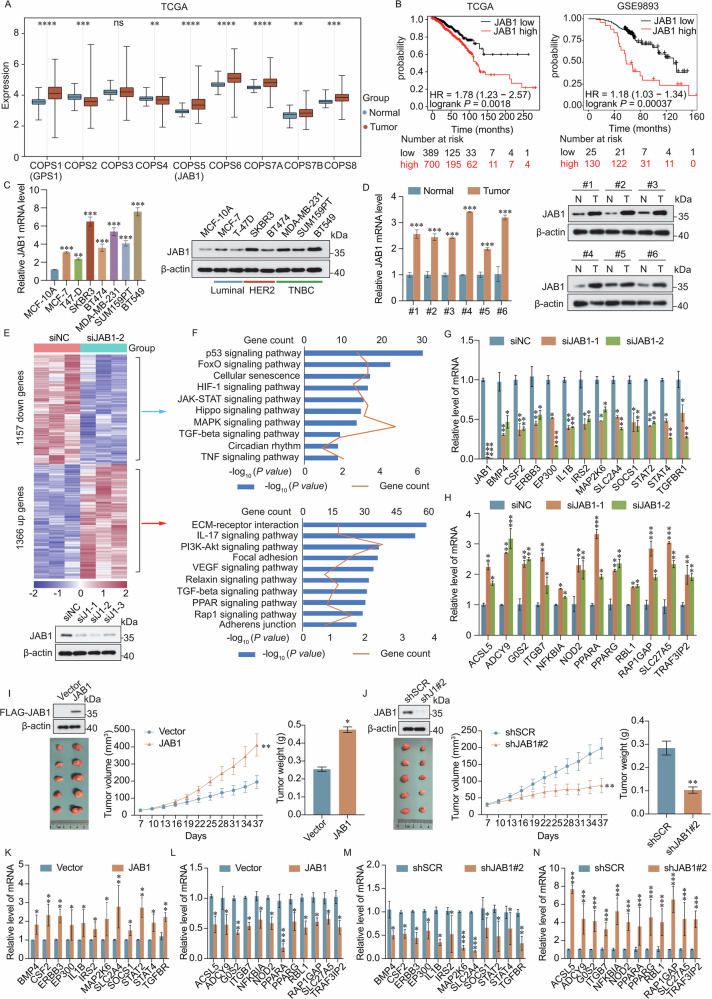
*JAB1* is an oncogene that promotes breast cancer progression. **A** The TCGA data presents the expression profiles of CSN family members in normal tissues and breast cancer tissues. **B** Prognostic potential of *JAB1* expression in breast cancer was analyzed using Kaplan–Meier survival analysis based on public datasets (TCGA and GSE9893). **C** *JAB1* mRNA and protein expression in normal mammary epithelial cells and breast cancer cell lines. **D** *JAB1* expression levels in breast cancer tissues versus adjacent normal tissues (*n* = 6). The patient-specific information is presented in Table S1. The patients included in this study were from the Cancer Hospital, Chinese Academy of Medical Sciences, and the relevant ethical approval number is: NCC2025C-302. **E** Heatmap of changes in the expression profiles between control (siNC) and JAB1-knockdown (siJAB1) MDA-MB-231 cells. **F** KEGG pathway enrichment was performed on the genes differentially expressed between control and JAB1-knockdown cells. **G**, **H** The expression levels of selected oncogenes and tumor suppressor genes in JAB1-knockdown MDA-MB-231 cells were assessed by qRT-PCR. **I**, **J** The efficiency of stable overexpression or knockdown of *JAB1* was assessed by western blotting. Subsequently, the MDA-MB-231 cells were injected into NOD-SCID mice (*n* = 5), and the tumor volume and weight were evaluated; J1, JAB1. **K**–**N** Using qRT-PCR technology, the expression levels of the aforementioned tumor suppressor genes and oncogenes were detected in tumors with *JAB1* overexpression or knockdown. Results are expressed as mean ± SD; two-tailed unpaired *t*-test; *p < 0.05, **p < 0.01, ***p < 0.001, ****p < 0.0001; ns not significant.

To investigate the function of the CSN in breast cancer, we analyzed clinical data from Gene Expression Omnibus (GEO) and The Cancer Genome Atlas (TCGA). Analysis of TCGA or GEO data revealed that multiple subunits of the COP9 signalosome (including GPS1/COPS1, COPS2, COPS4, JAB1/COPS5, COPS6, COPS7A, COPS7B, and COPS8) were significantly upregulated in breast cancer tissues compared with normal tissues (Fig. 1A and Supplementary Fig. 1A, B). Notably, while JAB1 expression in the TCGA cohort showed no significant association with molecular subtypes, analysis of the GSE65194 dataset identified its specific upregulation in triple-negative breast cancer (TNBC) patients, with no significant differences observed across HER2-positive, Luminal A, or Luminal B subtypes (Supplementary Fig. 1C, D). Survival analysis demonstrated that high expression of COPS4, JAB1 (COPS5), and COPS8 was significantly associated with reduced overall survival in patients (Fig. 1B). In contrast, high expression of COPS1, COPS6, COPS7A, COPS7B, and COPS8 also indicated poorer prognosis (Supplementary Fig. 1E, F). Collectively, these results suggest that JAB1, which is the focus of this investigation, is substantially upregulated in breast cancer and correlates closely with unfavorable patient outcomes. In line with these findings, *JAB1* was more highly expressed in various breast cancer cell lines than in normal breast epithelial cells MCF-10A (Fig. 1C). Furthermore, our newly collected samples, which included 6 pairs of breast cancer tissues and adjacent normal tissues, further demonstrated that JAB1 was significantly overexpressed in breast cancer (Fig. 1D). These findings suggest that *JAB1* overexpression is related to breast cancer initiation and progression.

To further explore the role of JAB1 in breast tumors, RNA-seq was performed on MDA-MB-231 cells treated with siRNA targeting JAB1. Compared to controls, 1366 genes were upregulated and 1157 downregulated in JAB1-knockdown cells (fold change ≥ 1.5; *p* < 0.05; Fig. 1E and Supplementary Fig. 2A). Kyoto Encyclopedia of Genes and Genomes (KEGG) pathway analysis revealed that differentially expressed genes (DEGs) regulated by *JAB1* were primarily enriched in cancer-related pathways, including p53 signaling, cellular senescence, HIF-1 signaling, TGF-beta signaling, IL-17 signaling and PPAR signaling, for both downregulated and upregulated genes (Fig. 1F). We validated the mRNA expression levels of relevant oncogenes and tumor suppressor genes in MCF-7 cells and MDA-MB-231 cells. Consistent with the RNA-seq analysis results, in JAB1-downregulated cells, the expression of oncogenes such as *BMP4, CSF2, ERBB3, EP300, IL1B, IRS2, MAP2K6, SLC2A4, SOCS1, STAT2, STAT4*, and *TGFBR1* decreased, while the expression of potential tumor suppressor genes, including *ACSL5, ADCY9, G0S2, ITGB7, NFKBIA, NOD2, PPARA, PPARG, RBL1, RAP1GAP, SLC27A5*, and *TRAF3IP2*, increased (Fig. 1G, H and Supplementary Fig. 2B, C).

To validate the results in vivo, we injected female NOD-SCID mice with MCF-7 cells and MDA-MB-231 cells overexpressing or knocking down JAB1. JAB1 overexpression enhanced tumor growth (Supplementary Fig. 2D and Fig. 1I), while knockdown had the opposite effect (Supplementary Fig. 2E and Fig. 1J). In orthotopically implanted breast tumors, oncogenes were upregulated in JAB1-overexpressing tissues and downregulated in JAB1-knockdown tissues (Supplementary Fig. 2F, H and Fig. 1K, M), while tumor suppressor genes showed the opposite pattern (Supplementary Fig. 2G, I and Fig. 1L, N). These results collectively demonstrate that JAB1 promotes breast cancer growth by coordinating the expression of vital tumor-regulating genes.

### JAB1 promotes breast cancer cell proliferation, invasion, and stemness

**Fig. 2:**
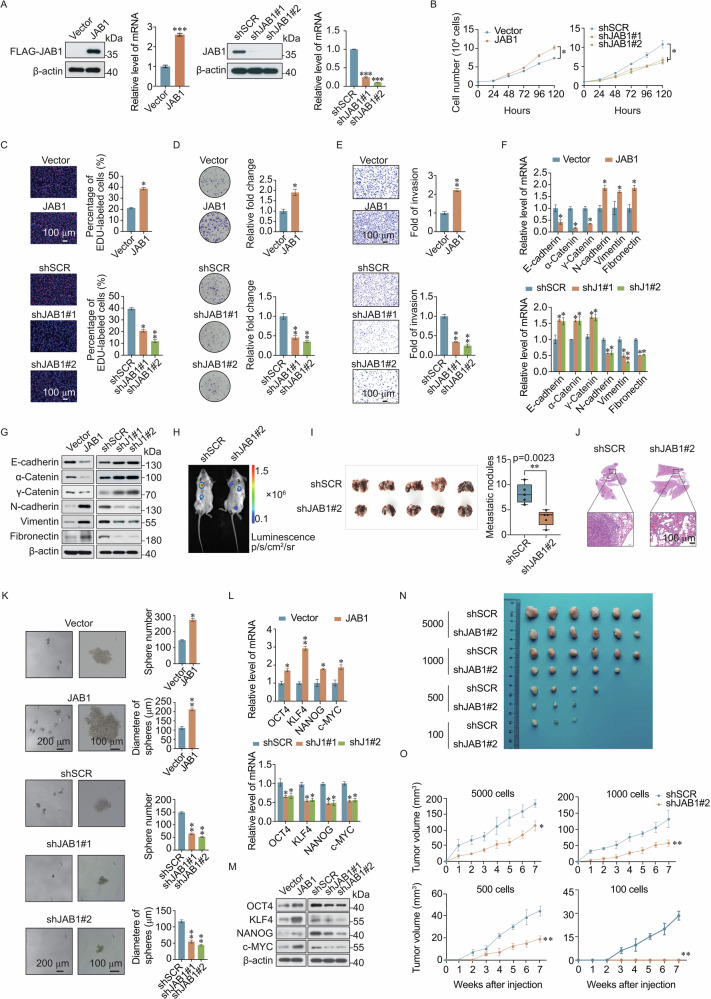
JAB1 promotes breast cancer cell proliferation, invasion, and stemness. **A** *JAB1* knockdown and overexpression efficiency in MDA-MB-231 cells assessed by qRT-PCR and western blotting. **B** Cell proliferation curves in MDA-MB-231 cells transfected with shSCR, shJAB1, Vector, or FLAG-JAB1. **C** Example images of EdU assays conducted on MDA-MB-231 cells following transfection with the respective lentiviral Vectors; Scale bar, 100 μm. **D** Representative images of colony formation assays in MDA-MB-231 cells transfected with the corresponding lentiviral Vectors. **E** Representative images of cell invasion assays performed with MDA-MB-231 cells using Matrigel Transwell chambers. Expression of epithelial and mesenchymal marker mRNA (**F**) and protein (**G**) in MDA-MB-231 cells; J1, JAB1. **H** Lung metastasis was observed by small animal imaging after tail vein injection of MDA-MB-231 cells. **I** Images of mouse lung nodules and statistical analysis. **J** H&E staining of mouse lung tissue. **K** Representative images of mammosphere formation assays in MDA-MB-231 cells show both the number (left panel, scale bar: 200 μm) and diameter (right panel, scale bar: 100 μm) of spheres after 15 days of culture. Expression of stemness markers mRNA (**L**) and protein (**M**) in MDA-MB-231 cells; J1, JAB1. **N** *JAB1* knockdown suppressed the stemness of breast cancer in vivo. MDA-MB-231 cells stably transfected with shSCR and shJAB1 were injected into the fourth pair of mammary fat pads of NOD/SCID female mice (*n* = 6 per group). **O** Tumor growth curves of NOD/SCID mice injected with shSCR or shJAB1 MDA-MB-231 cells at different cell doses. J1, JAB1; The error bars indicate the mean ± SD derived from three separate experiments; two-tailed unpaired *t*-test; *p < 0.05, **p < 0.01, ***p < 0.001.

To gain a clearer understanding of the function of JAB1 in breast tumors, we performed both overexpression and knockdown experiments. For the overexpression study, MCF-7 and MDA-MB-231 cells were infected with lentiviruses harboring the JAB1 coding sequence (CDS). In the knockdown experiment, two distinct short hairpin RNAs (shRNAs) were delivered to MCF-7 and MDA-MB-231 cells via lentivirus vectors to achieve stable JAB1 knockdown. JAB1 overexpression and knockdown efficiency were validated at the mRNA and protein levels (Supplementary Fig. 3A and Fig. 2A). Analysis of growth curves, EdU (5-ethynyl-2’-deoxyuridine) incorporation tests, and colony formation tests showed that cells overexpressing JAB1 exhibited a higher proliferation rate than controls, while cells with JAB1 knockdown experienced reduced growth (Supplementary Fig. 3B–D and Fig. 2B–D).

To evaluate JAB1’s role in breast cancer cell invasion and metastasis, we performed wound healing and transwell assays. JAB1 overexpression enhanced migration and invasion, while knockdown had opposite effects (Supplementary Fig. 3E, F and Fig. 2E). In MCF-7 and MDA-MB-231 cells, JAB1 overexpression downregulated epithelial markers (E-cadherin, α-catenin, γ-catenin) and upregulated mesenchymal markers (N-cadherin, vimentin, fibronectin) at mRNA and protein levels. Knockdown had opposite effects (Supplementary Fig. 3G, H and Fig. 2F, G). After tail vein injection of MDA-MB-231 cells, in vivo imaging of live small animals revealed more pronounced lung metastasis in the control group compared to the JAB1 knockdown group (Fig. 2H). Further quantification of lung nodules and HE staining confirmed these findings (Fig. 2I, J). EMT is reportedly involved in stem-like cell characteristic generation and maintenance [27, 28]. Therefore, we conducted mammosphere formation assays to explore the potential role of JAB1 in breast cancer cell stemness. JAB1 overexpression increased tumor sphere count and size, while knockdown decreased them (Supplementary Fig. 3I and Fig. 2K). Stemness markers (OCT4, KLF4, NANOG, c-MYC) were elevated with JAB1 overexpression and reduced with knockdown at mRNA and protein levels (Supplementary Fig. 3J, K and Fig. 2L, M). Results from limiting dilution transplantation assays in NOD/SCID mice demonstrated that JAB1 knockdown significantly reduced tumor-initiating capacity. Compared to the control group, the JAB1 knockdown group exhibited decreased breast cancer stem cell frequency (Fig. 2N). Tumors in the JAB1 knockdown group showed significantly slower growth rates, along with markedly reduced tumor volume and weight relative to the control group (Fig. 2O and Supplementary Fig. 3L). Overall, JAB1 promotes breast cancer cell proliferation, invasion, EMT, and stemness.

### JAB1 is physically associated with the CRL4B complex and stabilizes CUL4B

**Fig. 3:**
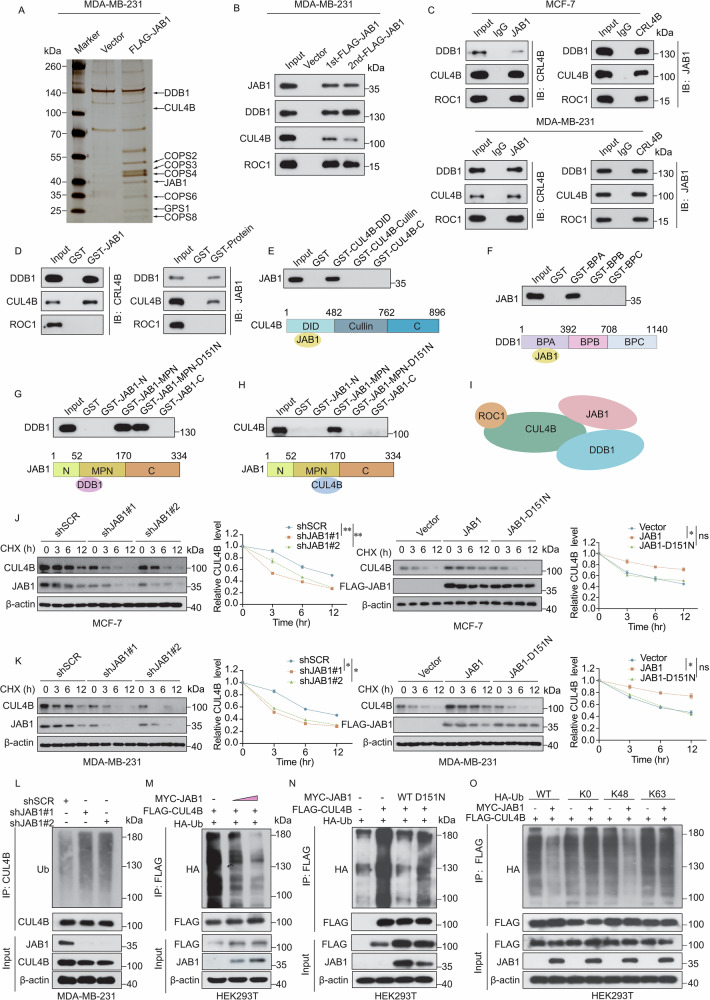
JAB1 is physically associated with the CRL4B complex and stabilizes CUL4B. **A** Immunoaffinity purification and mass spectrometry analysis were performed on JAB1-associated proteins in MDA-MB-231 cells. **B** The relevant fractions were validated by western blotting. **C** Co-immunoprecipitation analysis of the association between JAB1 and the CRL4B complex in MCF-7 and MDA-MB-231 cells. **D** GST pulldown assay of the interaction between JAB1 and CRL4B using GST fusion proteins. **E–H** Essential domains for the interactions between JAB1, DDB1, and CUL4B. **I** Schematic diagram of the molecular interactions between JAB1 and the CRL4B complex. **J**, **K** Expression of CUL4B in breast cancer cells with JAB1 knocked down or overexpressed (wild-type or mutant) and treated with cycloheximide (CHX, 50 μg/mL). **L** Ubiquitination status of CUL4B in JAB1-knockdown MDA-MB-231 cells. **M** Ubiquitination status of CUL4B in HEK293T cells co-transfected with FLAG-CUL4B, HA-tagged ubiquitin, and varying doses of MYC-JAB1. **N** The ubiquitination status of CUL4B was examined in HEK293T cells that were co-transfected with FLAG-CUL4B, HA-tagged ubiquitin, and either wild-type or mutant MYC-JAB1. **O** Ubiquitination status of CUL4B in HEK293T cells co-transfected with FLAG-CUL4B, MYC-JAB1, and HA-tagged ubiquitin mutants (K0: all lysine residues replaced by arginine; K48 and K63: all lysine residues except K48 or K63 are mutated to arginine). The error bars indicate the mean ± SD derived from three separate experiments; two-tailed unpaired *t*-test; *p < 0.05, **p < 0.01; ns, not significant.

Affinity purification and mass spectrometry analysis were conducted to identify potential JAB1 cofactor proteins. JAB1 co-purified with DDB1 and CUL4B (Fig. 3A and Table S2). The elution fractions from JAB1 mass spectrometry analysis validated the presence of JAB1, DDB1, CUL4B, and ROC1 (Fig. 3B). Co-immunoprecipitation (Co-IP) assays further verified the existence of the JAB1-associated protein complex. In MCF-7 and MDA-MB-231 cells, the CRL4B complex interacted with JAB1 (Fig. 3C and Supplementary Fig. 4A). Subsequently, glutathione S-transferase (GST) pull-down assays revealed that JAB1 directly interacts with both CUL4B and DDB1 (Fig. 3D). Additionally, JAB1 interacted with the DID domain of CUL4B and the BPA domain of DDB1, while DDB1 and CUL4B interacted with the MPN enzymatic domain of JAB1. However, point mutations in the MPN domain of JAB1 did not abolish its interaction with DDB1 (Fig. 3E–I and Supplementary Fig. 4B). Our experiments have revealed a direct interaction between JAB1 and CUL4B, suggesting a potential functional synergy between the two.

MDA-MB-231 cells with siRNA targeting JAB1 showed decreased CUL4B protein but unchanged mRNA (Supplementary Fig. 4C). Conversely, JAB1 overexpression increased CUL4B protein without affecting mRNA (Supplementary Fig. 4D). To assess proteasome dependence, cells were treated with MG132. This treatment partially reversed the CUL4B reduction in JAB1-knockdown cells, suggesting that additional degradation pathways for CUL4B may exist (Supplementary Fig. 4E). JAB1 overexpression in knockdown cells restored CUL4B levels (Supplementary Fig. 4F). CHX treatment increased CUL4B instability in JAB1-knockdown cells but enhanced stability with JAB1 overexpression (Supplementary Fig. 4G, H). The point mutations (specifically, the substitution of the critical aspartate residue at position 151 with asparagine, designated as JAB1–D151N) within JAB1’s enzymatic domain did not affect its interaction with CUL4B. Moreover, it was the wild-type JAB1, rather than the mutants, that extended CUL4B’s half-life (Supplementary Fig. 4I–K). Lentiviral transfection constructed JAB1 knockdown and overexpression in MCF-7 and MDA-MB-231 cells. Consistently, CHX treatment assays demonstrated that CUL4B instability was increased in knockdown cells and decreased in wild-type cells overexpressing JAB1 (Fig. 3J, K).

Based on the conserved JAB1/MPN/Mov34 metalloenzyme (JAMM) domain within JAB1 and its documented role in regulating protein stability, this study investigated its effects on CUL4B protein stability. In MDA-MB-231 cells with JAB1 knockdown, the polyubiquitination level of endogenous CUL4B was significantly increased (Fig. 3L). Overexpression of wild-type JAB1 in HEK293T cells significantly reduced polyubiquitination of exogenous CUL4B (Fig. 3M). In contrast, overexpression of the enzymatically inactive JAB1-D151N mutant attenuated, but did not fully abolish, this reduction (Fig. 3M, N). These findings suggest that JAB1 primarily maintains CUL4B stability via its catalytic domain, while the protein itself may also contribute through non-catalytic mechanisms. To determine the type of polyubiquitin chains on CUL4B, ubiquitination assays were performed using the mutant ubiquitin. JAB1 specifically cleaved the K48-linked ubiquitin chains on CUL4B (Fig. 3O and Supplementary Fig. 4L). Thus, JAB1 may maintain CUL4B stability in breast cancer.

### JAB1 promotes breast cancer progression by stabilizing CUL4B

**Fig. 4:**
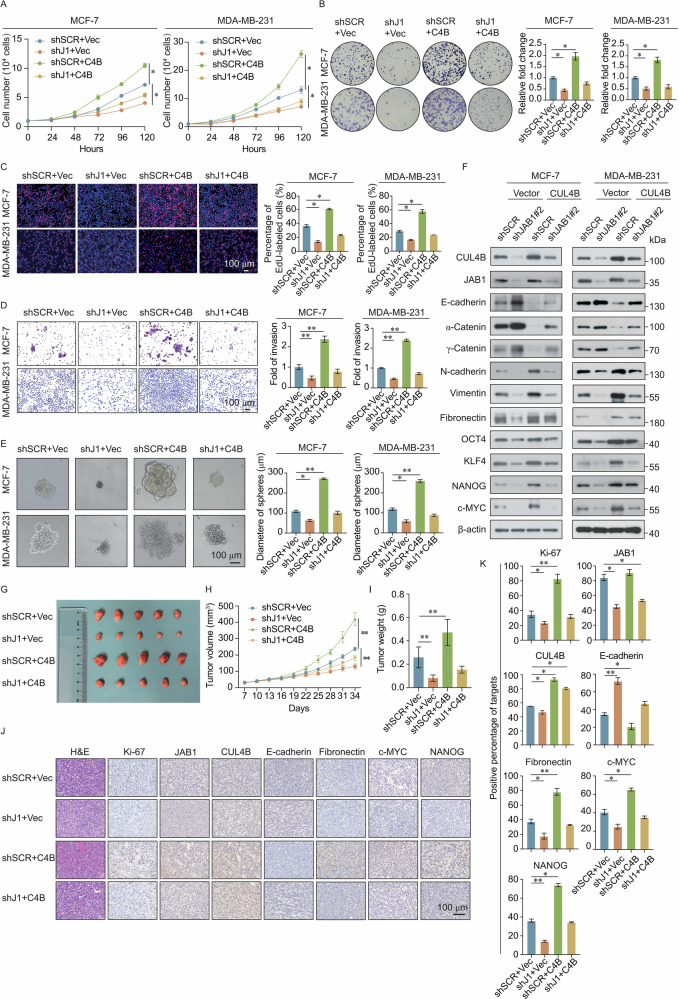
JAB1 promotes breast cancer progression by stabilizing CUL4B. **A** Proliferation curves were generated for MCF-7 and MDA-MB-231 cells following transfection with shSCR, shJAB1, empty Vector, or FLAG-CUL4B. **B** Representative images of colony formation assays in MCF-7 and MDA-MB-231 cells transfected with the corresponding lentiviral Vectors. **C** Illustrative images of EdU staining in MCF-7 and MDA-MB-231 cells following transfection with the appropriate lentiviral Vectors; scale bar, 100 μm. **D** Matrigel Transwell MCF-7 and MDA-MB-231 cell invasion assay. Images represent one microscopic field per group; scale bar, 100 μm. **E** Representative images of sphere diameter sizes in the mammary sphere formation assay; scale bar, 100 μm. **F** Abundance of epithelial, mesenchymal, and stemness markers. **G** NOD/SCID mice (*n* = 5) were implanted with MDA-MB-231 cells that had been transfected with shSCR, shJAB1, Vector control, or FLAG-CUL4B into their mammary gland fat pads. **H** Tumor growth in xenograft mice infected with different lentiviruses. **I** Tumor weights. **J**, **K** Immunohistochemical staining and analysis of Ki67, JAB1, CUL4B, E-cadherin, Fibronectin, c-MYC, and NANOG in tumor sections from xenograft mice infected with different lentiviruses. Vec Vector, J1 JAB1, C4B CUL4B. (**A**–**D**, **F**, **H**, **I**, **K**) The error bars indicate the mean ± SD derived from three separate experiments; two-tailed unpaired *t*-test; *p < 0.05, **p < 0.01.

Growth curve, colony formation, and EdU incorporation assays revealed that MCF-7 and MDA-MB-231 cell proliferation was inhibited by JAB1 knockdown and promoted by CUL4B overexpression. Moreover, the impact of CUL4B overexpression was reduced when JAB1 was knocked down (Fig. 4A–C). Meanwhile, concurrent knockdown of JAB1 and CUL4B resulted in a more pronounced reduction in cell proliferation. Conversely, their simultaneous overexpression led to a more than twofold increase in proliferation, while the effect of JAB1 overexpression was attenuated by CUL4B knockdown (Supplementary Fig. 5A).

Transwell assays showed that JAB1 knockdown inhibited breast cancer cell invasion, while CUL4B overexpression promoted it. Simultaneous knockdown of both reduced invasion more than single gene knockdown; concurrent overexpression enhanced invasion by more than twofold. The effects of one gene’s overexpression were attenuated by the other’s knockdown (Supplementary Fig. 5B and Fig. 4D). In JAB1-knockdown cells, epithelial markers increased and mesenchymal markers decreased; CUL4B overexpression had opposite effects, which were diminished by JAB1 knockdown (Fig. 4E). Mammosphere assays revealed that JAB1 knockdown reduced and CUL4B overexpression increased tumor sphere diameter. Simultaneous knockdown decreased the diameter more than single gene knockdown; concurrent overexpression increased it more pronouncedly. Again, effects were mitigated by knockdown of the other gene (Supplementary Fig. 5C and Fig. 4F). JAB1 knockdown also reduced stemness markers, which CUL4B overexpression increased, but this effect was weakened by JAB1 knockdown (Fig. 4E).

In vivo, JAB1 knockdown significantly reduced subcutaneous tumor growth in mice, while CUL4B overexpression significantly increased growth. Additionally, JAB1 knockdown mitigated the promoting effect of CUL4B overexpression on tumor growth (Fig. 4G–I). Immunohistochemistry (IHC) results demonstrated that, compared with the control group, JAB1 knockdown significantly reduced the expression levels of Ki67, JAB1, CUL4B, Fibronectin, c-MYC, and NANOG, while increasing E-cadherin expression. Meanwhile, CUL4B overexpression elevated the levels of Ki67, CUL4B, Fibronectin, c-MYC, and NANOG, and decreased E-cadherin expression; these effects were reversed by JAB1 knockdown (Fig. 4J, K). Collectively, these findings indicate that JAB1 enhances breast cancer cell growth, invasiveness, and stem cell-like properties through the stabilization of CUL4B protein.

### Combined JAB1 inhibitors and chemotherapeutic drugs synergistically reverse drug resistance in breast cancer cells

**Fig. 5:**
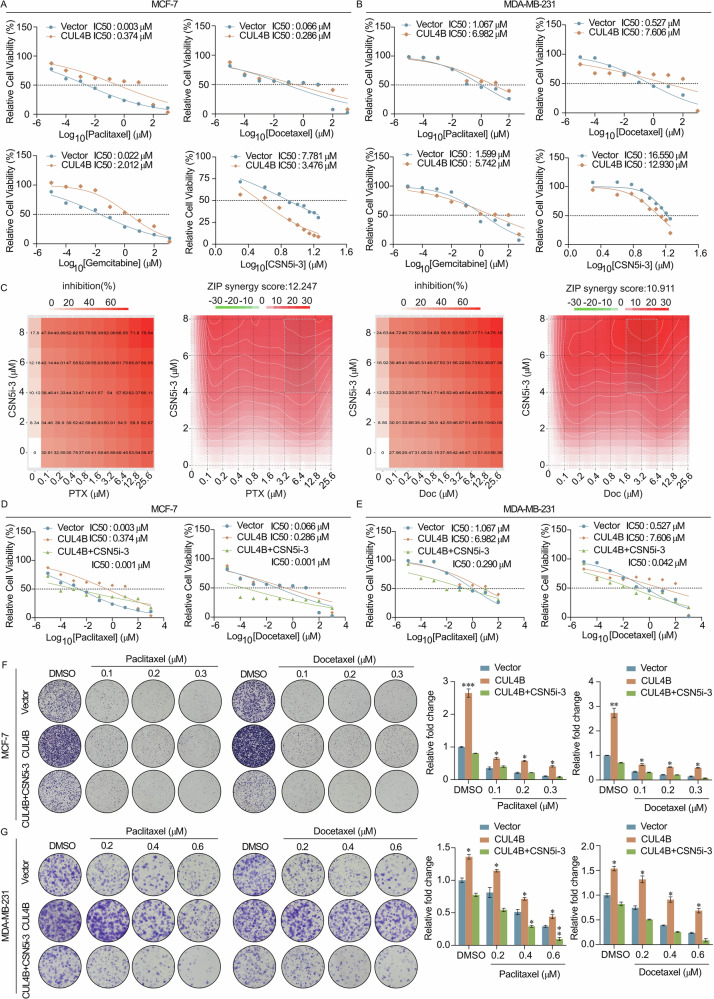
Combining JAB1 inhibitors and chemotherapeutic drugs synergistically reverses drug resistance in breast cancer cells. **A**, **B** In cells transfected with lentiviral Vector or FLAG-CUL4B, continuous doses of paclitaxel, docetaxel, gemcitabine, or CSN5i-3 were added, and the IC_50_ values of each drug were determined by CCK-8 assay. **C** Impact of combined CSN5i-3 and paclitaxel or docetaxel on MDA-MB-231 cells, presented as dose-response matrices and synergy score matrices. **D**, **E** In cells transfected with lentiviral Vector or FLAG-CUL4B, the lowest effective dose of CSN5i-3 was added along with varying doses of paclitaxel or docetaxel. The cells were then assayed using the CCK-8 test. **F**, **G** The clone formation assay was conducted in breast cancer cells transfected with either a lentiviral Vector or FLAG-CUL4B following treatment with the minimum effective dose of CSN5i-3 in combination with varying concentrations of paclitaxel or docetaxel. (**A**, **B**, **D**) The error bars indicate the mean ± SD derived from three separate experiments; two-tailed unpaired *t*-test; (**F**, **G**) two-way ANOVA; *p < 0.05, **p < 0.01, ***p < 0.001.

To further investigate how JAB1 regulates CUL4B to promote breast cancer progression, we evaluated the therapeutic efficacy of five chemotherapeutic drugs (paclitaxel, docetaxel, epirubicin, doxorubicin, and gemcitabine) in breast cancer cells transfected with either a control Vector or FLAG-CUL4B. CUL4B overexpression increased the half-maximal inhibitory concentration (IC_50_) of paclitaxel, docetaxel, gemcitabine, and doxorubicin in both MCF-7 and MDA-MB-231 cells. Meanwhile, the sensitivity to the JAB1 inhibitor CSN5i-3 was significantly enhanced in CUL4B-overexpression cells compared to the control group (Fig. 5A, B and Supplementary Fig. 6A–C). Therefore, combining a JAB1 inhibitor with chemotherapeutic drugs (paclitaxel, docetaxel, doxorubicin, and gemcitabine) might effectively inhibit breast cancer progression.

The ZIP model in Synergy Finder 2.0 revealed that combined paclitaxel or docetaxel with the JAB1 inhibitor CSN5i-3 demonstrated significant drug synergy; the optimal dosages of each agent in the combined regimen were further determined (Fig. 5C and Supplementary Fig. 6D). Additionally, treatment of MCF-7 and MDA-MB-231 cells overexpressing CUL4B with the minimum effective dose of CSN5i-3 (4 μM) significantly reduced the IC_50_ of paclitaxel and docetaxel (Fig. 5D, E). Hence, silencing JAB1 sensitized breast cancer cells to taxane-based chemotherapy.

Colony formation assays showed that treatment of MCF-7 and MDA-MB-231 cells overexpressing CUL4B with CSN5i-3 (4 μM) significantly enhanced the inhibitory effect of chemotherapeutic drugs on tumor proliferation (Fig. 5F, G). Combining CSN5i-3 with paclitaxel or docetaxel also significantly reduced the invasive ability of the cells (Supplementary Fig. 6E, F). In summary, the small molecule inhibitor CSN5i-3, which targets JAB1, can reverse the chemotherapy resistance caused by high CUL4B expression in breast cancer cell lines.

### The JAB1/CRL4B Complex Co-Transcriptionally Represses the Expression of PPARG and ACSL5

**Fig. 6:**
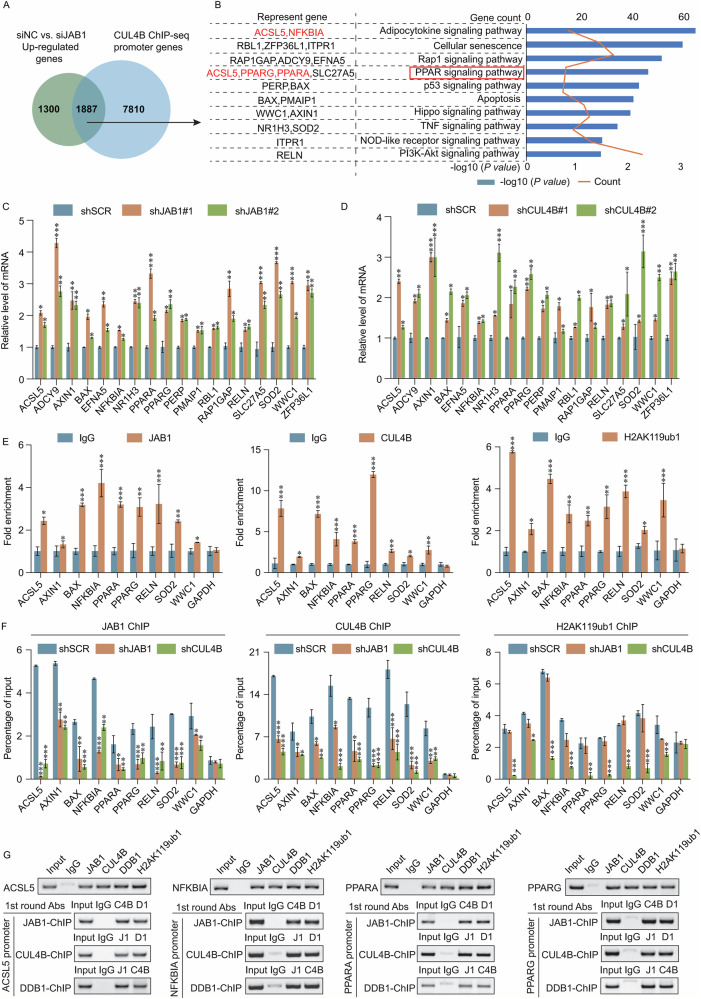
The JAB1/CRL4B complex co-transcriptionally represses the expression of PPARG and ACSL5. **A** Venn diagram of upregulated genes from JAB1 RNA-seq and promoter genes from CUL4B ChIP-seq. **B** KEGG pathway analysis of the 1887 overlapping upregulated genes from JAB1 RNA-seq data and the promoter genes from CUL4B ChIP-seq data. **C** qRT-PCR analysis of mRNA expression of target genes in JAB1-knockdown MDA-MB-231 cells. **D** qRT-PCR analysis of mRNA expression of target genes in CUL4B-knockdown MDA-MB-231 cells. **E** qChIP analysis of the identified genes in MDA-MB-231 cells. **F** qChIP analysis of MDA-MB-231 cells on the promoters of target genes using antibodies against JAB1, CUL4B, or H2K119ub1. Results are expressed as fold change (FC) relative to control, with GAPDH as the negative control. **G** ChIP and Re-ChIP experiments in MDA-MB-231 cells. J1 JAB1, C4B CUL4B, D1 DDB1. (**C**–**F**) The error bars indicate the mean ± SD derived from three separate experiments; two-tailed unpaired *t*-test; *p < 0.05, **p < 0.01, ***p < 0.001.

To further demonstrate that the JAB1/CRL4B complex can jointly regulate the progression of breast cancer, we evaluated the upregulated genes in the JAB1 RNA-seq data (fold change ≥ 1.2; *p* < 0.001) and the promoter genes identified in the CUL4B ChIP-seq data (*p* < 0.001), and screened out 1,887 overlapping genes between the two datasets (Fig. 6A). An analysis using KEGG indicates that JAB1 and CUL4B share potential targets that participate in cellular senescence, along with the adipocytokine, PPAR, p53, and TNF signaling pathways (Fig. 6B). After knocking down JAB1 or CUL4B in MDA-MB-231 cells, qRT-PCR analysis demonstrated a marked upregulation in the expression of genes that are enriched in the pathways identified by KEGG (Fig. 6C, D). Quantitative ChIP (qChIP) analysis further confirmed that JAB1 and CUL4B are significantly enriched at the promoter regions of multiple tumor suppressor genes [29–46], including *ACSL5*, *AXIN1*, *BAX*, *NFKBIA*, *PPARA*, *PPARG*, *RELN*, *SOD2*, and *WWC1* (Fig. 6E).

Recruitment to the promoters of these target genes was decreased in JAB1- or CUL4B-knockdown MDA-MB-231 cells. To determine whether the JAB1/CRL4B complex mediates transcriptional repression by catalyzing H2AK119ub1, our qChIP analysis revealed that in MDA-MB-231 cells. However, recruitment of the complex to target promoters decreased following JAB1 and CUL4B knockdown; H2AK119ub1 levels remained largely unchanged after JAB1 depletion (Fig. 6F). This suggests that the JAB1/CRL4B complex may mediate transcriptional repression through a mechanism independent of H2AK119ub1 mark deposition. Subsequent ChIP/Re-ChIP experiments were conducted on four candidate target genes: *ACSL5, NFKBIA, PPARA*, and *PPARG* (Fig. 6G). The promoters of *ACSL5, NFKBIA, PPARA*, and *PPARG* which were immunoprecipitated with the JAB1 antibody, were then re-immunoprecipitated with the CUL4B or DDB1 antibody. Therefore, JAB1 associates with the CRL4B complex to jointly transcriptionally repress these target genes.

### JAB1 and CUL4B Enhance the Proliferation and Invasion of Breast Cancer Cells, and Induce Fatty Acid Metabolic Reprogramming, through the Inhibition of PPARG/ACSL5 Expression

**Fig. 7:**
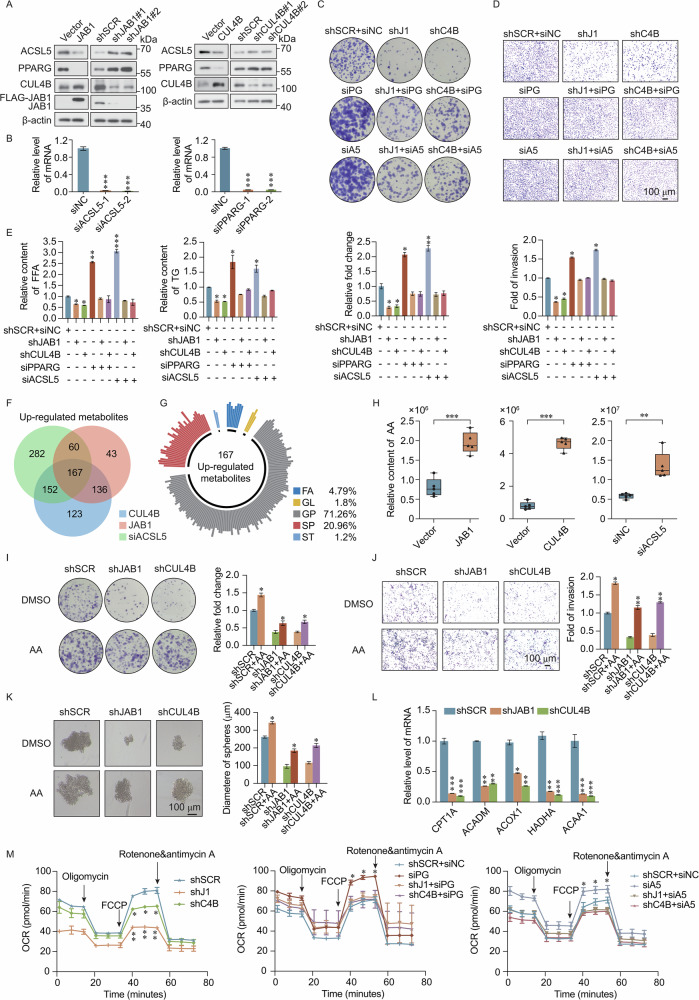
JAB1 and CUL4B enhance the proliferation and invasion of breast cancer cells, and induce fatty acid metabolic reprogramming, through the inhibition of PPARG/ACSL5 expression. **A** The protein expression levels of ACSL5 and PPARG were detected in MDA-MB-231 cells with JAB1 knockdown or overexpression (left panel). The protein expression levels of ACSL5 and PPARG were also detected in MDA-MB-231 cells with CUL4B knockdown or overexpression (right panel). **B** The efficiency of ACSL5 or PPARG knockdown in MDA-MB-231 cells was confirmed using qRT-PCR. **C** Clone formation assays were conducted in MDA-MB-231 cells after transfection with control, shJAB1, shCUL4B, siPPARG, or siACSL5. **D** Transwell assays were conducted on MDA-MB-231 cells with control, shJAB1, shCUL4B, siPPARG, or siACSL5 transfection. **E** The relative levels of serum-free free fatty acids (FFA) and serum-free triglycerides (TG) were detected in MDA-MB-231 cells transfected with control, shJAB1, shCUL4B, siPPARG, or siACSL5. **F**, **G** Wide-targeted lipidomics analysis revealed upregulated lipid species and their distribution in MDA-MB-231 cells with JAB1 overexpression, CUL4B overexpression, and ACSL5 knockdown. **H** Wide-targeted lipidomics analysis results revealed the levels of arachidonic acid (AA) in each group. **I** Clone formation assays were performed in MDA-MB-231 cells (shSCR, shJAB1, and shCUL4B groups) following supplementation with 15 μM arachidonic acid. **J** Transwell assays were conducted in MDA-MB-231 cells (shSCR, shJAB1, and shCUL4B groups) following supplementation with 15 μM arachidonic acid. **K** Sphere formation assays were performed in MDA-MB-231 cells (shSCR, shJAB1, and shCUL4B groups) following supplementation with 15 μM arachidonic acid. **L** The expression levels of metabolites involved in fatty acid oxidation were detected by qRT-PCR in MDA-MB-231 cells. **M** Mitochondrial oxygen consumption rate (OCR) was examined in MDA-MB-231 cells transfected with control, shJAB1, shCUL4B, siPPARG, or siACSL5. J1, JAB1; C4B, CUL4B; PG, PPARG; A5, ACSL5. The error bars indicate the mean ± SD derived from three separate experiments; two-tailed unpaired *t*-test, *p < 0.05, **p < 0.01, ***p < 0.001.

To investigate whether JAB1 and CUL4B affect cell proliferation and invasion by transcriptionally repressing target genes, western blot analysis was first performed to detect the protein expression levels of the target genes in MCF-7 and MDA-MB-231 cells with stable JAB1 knockdown or overexpression. The results revealed a negative correlation between JAB1 expression and the expression of PPARG and ACSL5. Similar results were observed in cells with stable CUL4B knockdown or overexpression (Supplementary Fig. 7A and Fig. 7A). Subsequently, the knockdown efficiency of PPARG and ACSL5 in MCF-7 and MDA-MB-231 cells was validated (Supplementary Fig. 7B and Fig. 7B). Colony formation assays showed that the loss of function of PPARG or ACSL5 nearly rescued the inhibited cell proliferation caused by JAB1 and CUL4B knockdown (Supplementary Fig. 7C and Fig. 7C). PPARG or ACSL5 knockdown exerted a similar rescue effect on cell invasion (Supplementary Fig. 7D and Fig. 7D).

PPARG, a nuclear receptor, is vital for adipogenesis, insulin sensitivity, and inflammation, and regulates FASN, affecting fatty acid synthesis [47, 48]. ACSL5 catalyzes the formation of acyl-CoA from long-chain fatty acids, a crucial step for fatty acid metabolism [49, 50]. Therefore, the free fatty acid (FFA) and triglyceride (TG) content were measured in MCF-7 and MDA-MB-231 cells. Knockdown of JAB1 or CUL4B decreased FFA and TG levels in the cells, whereas knockdown of PPARG or ACSL5 had the opposite effect. Moreover, knocking down PPARG or ACSL5 rescued the reduced FFAs and TGs caused by JAB1 or CUL4B knockdown (Supplementary Fig. 7E and Fig. 7E). Given the crucial roles of ACSL5 and PPARG in fatty acid metabolism, we propose that the JAB1/CUL4B-ACSL5/PPARG axis may influence epithelial-mesenchymal transition (EMT) and stemness in breast cancer by regulating lipid metabolism. Consequently, we conducted wide-target lipidomic sequencing to detect changes in the levels of various fatty acids in breast cancer cells with overexpression of JAB1 and CUL4B as well as silencing of ACSL5. The results revealed that overexpression of JAB1 and CUL4B, combined with ACSL5 knockdown, collectively regulated the elevation of 167 lipid species within cells (Fig. 7F), including 8 free fatty acids, accounting for 4.79% (Fig. 7G). Arachidonic acid (AA), an essential component of cell membrane phospholipids, is involved in regulating inflammatory processes and cell signal transduction in the body. It showed a significant increase in all three groups with overexpression of JAB1 and CUL4B as well as ACSL5 knockdown (Supplementary Fig. 7F and Fig. 7H). Subsequently, we supplemented AA in breast cancer cells with JAB1 or CUL4B knockdown. We observed that the addition of exogenous arachidonic acid could reverse the reduced proliferative capacity of breast cancer cells caused by JAB1 or CUL4B knockdown (Supplementary Fig. 7G and Fig. 7I). Similarly, it could also reverse the diminished metastatic ability of breast cancer cells resulting from JAB1 or CUL4B knockdown (Supplementary Fig. 7H and Fig. 7J). In sphere-formation assays, arachidonic acid supplementation also significantly counteracted the decreased sphere-forming ability of breast cancer cells induced by JAB1 or CUL4B knockdown (Supplementary Fig. 7I and Fig. 7K). Meanwhile, we also examined the production of relevant metabolites in the fatty acid metabolic pathway. The qPCR experimental results indicated that knocking down JAB1 or CUL4B led to a decrease in the mRNA expression levels of downstream metabolic genes involved in fatty acid oxidation (Supplementary Fig. 7J and Fig. 7L). Additionally, the Seahorse XFe96 extracellular flux analyzer was employed to determine the oxygen consumption rate (OCR) of the cells. Knocking down JAB1 or CUL4B reduced the OCR in breast cancer cells, while knocking down PPARG or ACSL5 increased OCR and rescued the reduction caused by JAB1 or CUL4B knockdown (Supplementary Fig. 7K and Fig. 7M). Hence, breast cancer cells with PPARG or ACSL5 knockdown exhibited enhanced mitochondrial respiration. As upstream regulatory molecules, the loss of function of JAB1 or CUL4B severely impaired mitochondrial oxidative phosphorylation. Collectively, these results suggest that JAB1 and CUL4B regulate breast cancer occurrence and development by transcriptionally inhibiting PPARG/ACSL5, impacting fatty acid metabolism.

### JAB1 is overexpressed in various cancer types and serves as a prospective cancer biomarker

**Fig. 8:**
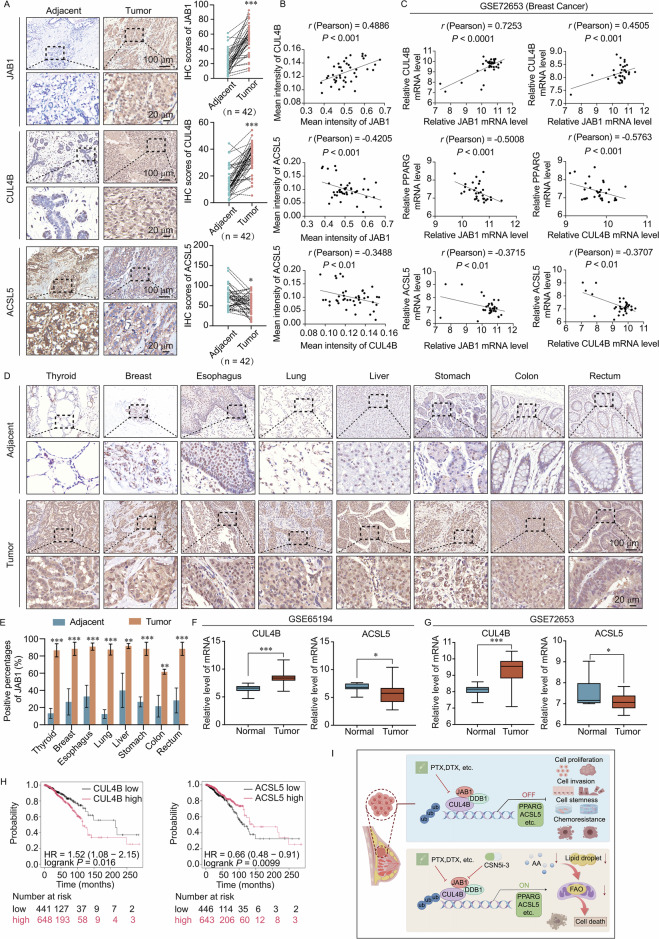
JAB1 is overexpressed in various cancer types and serves as a prospective cancer biomarker. **A**, **B** Immunohistochemical staining and expression analysis of JAB1, CUL4B, and ACSL5 in breast cancer and adjacent tissues (*p < 0.05, ***p < 0.001; two-tailed unpaired *t*-test). **C** Correlation analysis of JAB1, CUL4B, PPARG, and ACSL5 expression in a published clinical dataset (GSE72653) using a two-tailed unpaired *t*-test. **D**, **E** Immunohis*t*ochemical staining and analysis of JAB1 in matched tumor and adjacent normal tissues. Results are expressed as mean ± SD (**p < 0.01, ***p < 0.001; two-tailed unpaired *t*-test). **F**, **G** mRNA expression analysis of *CUL4B* or *ACSL5* in normal and breast cancer tissues using published clinical datasets (GSE65194, GSE72653). Results are expressed as mean ± SD (*p < 0.05, ***p < 0.001; two-tailed unpaired *t*-test). **H** Correlation analysis between *CUL4B* or *ACSL5* expression and survival of patients with breast cancer using a published clinical dataset (TCGA BRCA). **I** A proposed mechanism by which the JAB1/CUL4B complex modulates the initiation and progression of breast cancer via the PPARG/ACSL5 pathway. PTX paclitaxel, DTX docetaxel.

To explore the correlated expression of JAB1, CUL4B, and ACSL5 in breast cancer, IHC staining was performed to assess their protein levels in both breast cancer tissues and normal breast tissues. JAB1 and CUL4B levels were found to be increased in breast cancer samples, whereas ACSL5 expression was higher in normal breast tissues compared to breast cancer tissues (Fig. 8A). Thus, a positive correlation was observed between JAB1 and CUL4B expression, whereas a negative correlation was noted with ACSL5 expression in breast cancer tissues (Fig. 8B). Consistent results were obtained from the breast cancer dataset (GSE72653) (Fig. 8C).

To investigate whether the oncogenic potential of JAB1 extends to other cancer types, tissue microarray analyses were conducted using IHC staining. High JAB1 expression was detected in various cancer samples, including thyroid, breast, esophageal, lung, liver, gastric, colon, and rectal cancer, when compared with matched normal tissues (Fig. 8D, E). The relationship between JAB1, CUL4B, and their downstream target genes was further evaluated using TCGA and GEO datasets. Increased CUL4B expression and decreased ACSL5 expression were observed in breast cancer tissues (Fig. 8F, G). Furthermore, TCGA breast cancer data revealed that patients with high CUL4B expression had lower survival rates, while those with high ACSL5 expression exhibited significantly higher survival rates (Fig. 8H). In conclusion, JAB1 may act as an oncogene in multiple cancers, including breast cancer, thus underscoring its potential as a biomarker. A proposed mechanism by which the JAB1/CUL4B complex modulates the initiation and progression of breast cancer via the PPARG/ACSL5 pathway are described in Fig. 8I.

## Discussion

The results of this study indicate that JAB1 directly interacts with the CRL4B complex to stabilize CUL4B, thereby transcriptionally repressing the fatty acid metabolism-related genes PPARG and ACSL5, which ultimately promotes breast cancer progression (Fig. 8I). Accordingly, the oncogenic mechanism of the JAB1/CUL4B complex represents a potential therapeutic target.

JAB1, as a multifunctional protein molecule, participates in numerous biological functions, including cell proliferation and apoptosis, DNA damage repair, cellular senescence, chemotherapy resistance, and cell metabolism [51, 52]. The conserved JAMM (JAB1/MPR1P and PaD1P N-terminal (MPN) domain metalloenzyme motif is found in JAB1, which is the fifth component of the CSN [53]. JAMM is pivotal in the deubiquitination process within the CSN family [8]. Through its deubiquitinating activity, JAB1 facilitates the transport of p27kip1 from the nucleus to the cytoplasm and its degradation via the ubiquitin–proteasome pathway. This relieves the inhibitory effect of p27kip1 on CDK-cyclin complexes, promoting cell cycle progression and enhancing tumor cell proliferation [54]. Similarly, via deubiquitinating β-catenin and PD-L1 via JAB1, Protein Disulfide Isomerase Family A Member 6 (PDIA6) encourages the growth of pancreatic cancer and immune evasion [12]. Notably, our research has also demonstrated that overexpression of JAB1 not only promotes breast cancer cell proliferation but also enhances its invasive ability, EMT, and confers stem-like properties to cells. Therefore, the importance of JAB1 as a key oncogenic target in tumors is self-evident. In the future, we need more in-depth research to uncover the specific roles and molecular mechanisms of JAB1 in tumor progression, providing a theoretical basis for the development of effective antitumor strategies.

The CRL4B complex is a Cullin-Ring E3 ubiquitin ligase with transcriptional repressor activity via H2AK119 monoubiquitination. It’s abnormally expressed in various tumors. Indeed, we previously reported that RUNX2 interacts with the CRL4B complex, promoting the invasive capacity of breast cancer cells and the EMT phenotype [55]. Similarly, SIRT1 interacts with the CRL4B complex, promoting stem-like characteristics in pancreatic cancer cells [56]. In the current study, JAB1 was shown to directly bind CUL4B and DDB1 within the CRL4B complex, ultimately regulating the stability of CUL4B. The JAB1/CRL4B complex can promote breast cancer cell proliferation, invasion, and stemness. Experimental data indicate that this stabilization depends on JAB1’s catalytic MPN domain. It should be noted that mutation of JAB1’s catalytic domain not only affects its potential deubiquitination activity but also stops its deNEDDylation activity [8, 9]. Such impairment would not only inhibit CRL4B complexes activity and prevent its autoubiquitination but would also disrupt the ubiquitination processes of CRL components. Additionally, in bladder cancer, CRL4B inhibits miR-372/373 transcription, leading to upregulated PIK3CA and AKT activation, conferring chemotherapy resistance to cancer cells [57]. Similarly, our findings indicate that high expression of CUL4B leads to chemoresistance in breast cancer cells, but the JAB1 inhibitor CSN5i-3 enhances sensitivity. Thus, the JAB1/CRL4B complex is a potential breast cancer treatment target.

In breast cancer cells, fatty acid metabolism pathways are often abnormally activated. For instance, FASN is linked to tumor malignancy and prognosis and is significantly expressed in breast cancer [17]. Adipocytes within the breast tumor microenvironment can affect cancer cell fatty acid metabolism by secreting fatty acids and other substances [58]. This study demonstrates that JAB1 and CUL4B promote breast cancer initiation and progression by transcriptionally suppressing the expression of PPARG and ACSL5, key genes involved in fatty acid metabolism. The JAB1/CRL4B complex-mediated repression of these genes concurrently enhances both fatty acid synthesis and oxidation—a paradoxical phenomenon termed “metabolic coupling” which represents a hallmark of aggressive cancers[19, 59]. The underlying mechanism may involve spatial separation of antagonistic pathways: de novo synthesis in the cytoplasm and β-oxidation in mitochondria, enabling a “synthesis-for-growth” paradigm while sustaining low-level oxidation for bioenergetics[17, 60]. Alternatively, dual gene repression may lead to uncontrolled metabolic flux due to loss of regulatory restraint, wherein PPARG loss disrupts metabolic homeostasis and ACSL5 suppression causes dysregulated fatty acid channeling. Ultimately, within the oncogenic context, the JAB1/CRL4B complex acts as a master regulator of this plastic, coupled metabolic state, establishing a high-flux, adaptable network that supports rapid tumor proliferation and survival.

In this study, exogenous arachidonic acid (AA) supplementation promoted the proliferation, invasion, and stemness maintenance of breast cancer cells. AA, a key component of membrane phospholipids and a precursor to bioactive mediators, exerts pro-tumorigenic effects that are tissue-specific and dependent on the metabolic microenvironment [61, 62]. Although AA metabolic homeostasis does not initiate tumorigenesis under healthy physiological conditions, multiple studies have shown that AA metabolic dysregulation can promote tumor progression through multidimensional mechanisms [63, 64]. In our research, the JAB1/CRL4B complex, by suppressing metabolic genes including ACSL5, disrupted lipid homeostasis, thereby enhancing the utilization of exogenous AA and ultimately supplying ample biosynthetic precursors and energy to fuel tumor growth.

ACSL5 was found to impact fatty acid oxidation in breast cancer tumor cells within the current study. This is a key step in fatty acid metabolism, including oxidation, synthesis, and the synthesis of TGs and phospholipids [29, 50]. Interestingly, PPARG ligands, such as troglitazone and rosiglitazone, can downregulate the expression of JAB1 in hepatocellular carcinoma cells [65]. JAB1 also has a regulatory role in fatty acid metabolism by blocking ERK1/2 activation induced by palmitic acid (PA). Additionally, inhibiting JAB1 can promote lipid metabolism, boost hepatic glucose uptake, and reduce the production of inflammatory factors [66]. In the present study, we showed that the small-molecule inhibitor of JAB1, CSN5i-3, effectively inhibited the expression of JAB1. Although we did not further explore the combined therapeutic effect of PPARG ligands and the JAB1 small-molecule inhibitor CSN5i-3, we found that the combination of JAB1 inhibitors and chemotherapeutic drugs significantly increased the sensitivity of breast cancer cells with high CUL4B expression to chemotherapeutic agents.

Although the overall mechanisms of the JAB1/CRL4B complex remain not fully understood, this study suggests that the JAB1/CRL4B complex transcriptionally represses the fatty acid metabolism-related genes PPARG and ACSL5, thereby inducing fatty acid metabolic reprogramming, which ultimately enhances tumor cell growth and survival. Therefore, the complex comprising JAB1 and CRL4B may represent a potential oncogenic target in breast cancer, and the utilization of JAB1 inhibitors in conjunction with clinical chemotherapy drugs could potentially enhance the antitumor therapeutic efficacy.

## Supplementary information

Supplementary material file Figures

Supplementary material file Legends

Supplementary material file Tables

Supplementary material-Uncropped Western Blots

Supplementary material- Raw qPCR Data

## Data Availability

The raw data for the RNA-seq (siJAB1) in this study have been uploaded to the Gene Expression Omnibus (GEO) under the accession number GSE288090.
