# Supplementary figures and images for: JAB1/CRL4B complex represses PPARG/ACSL5 expression to promote breast tumorigenesis
Source: Cell Death Differ. 2025 Dec 12;33(6):1175–91. doi: 10.1038/s41418-025-01642-0 (PMC13247160; doi:10.1038/s41418-025-01642-0)

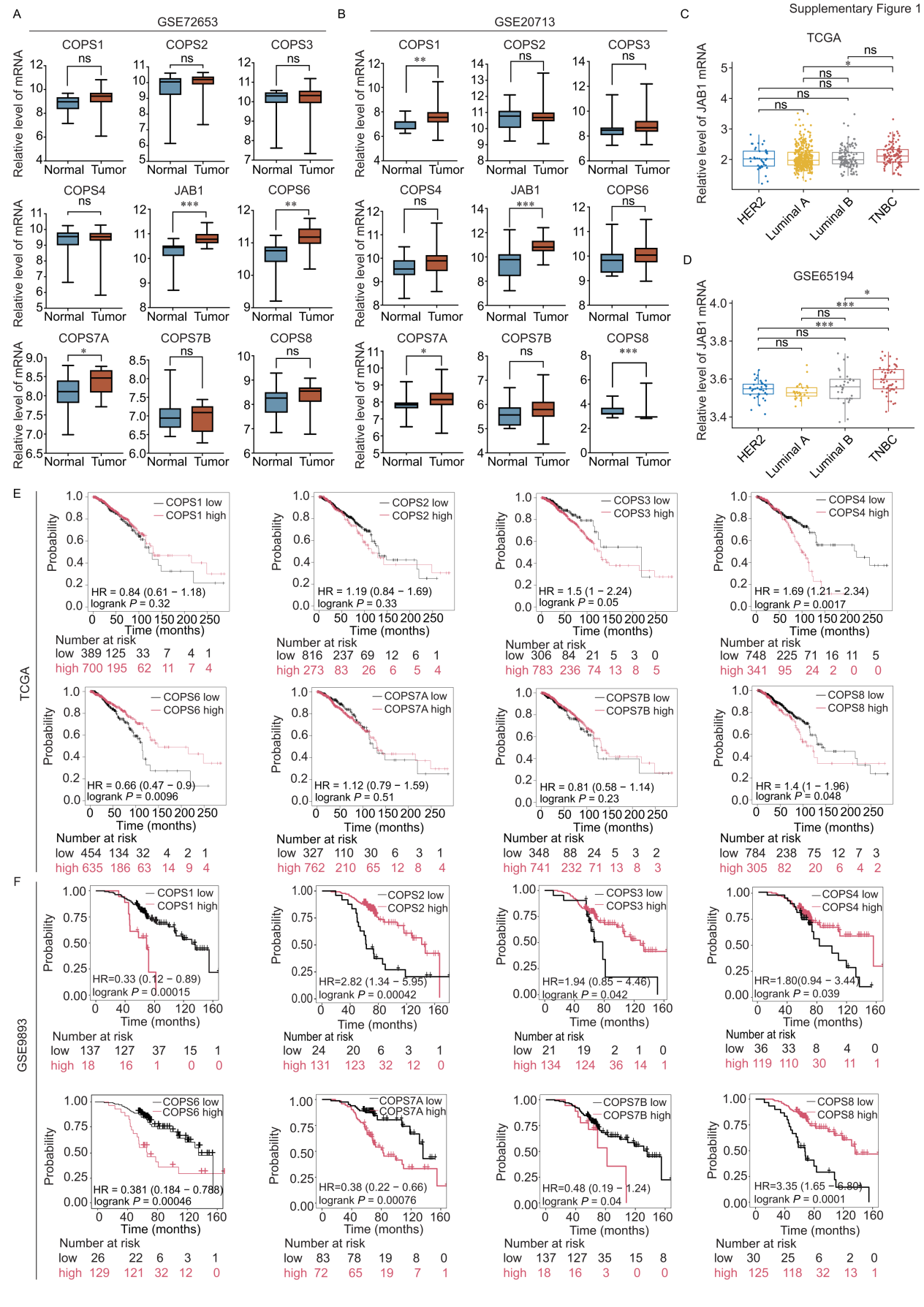


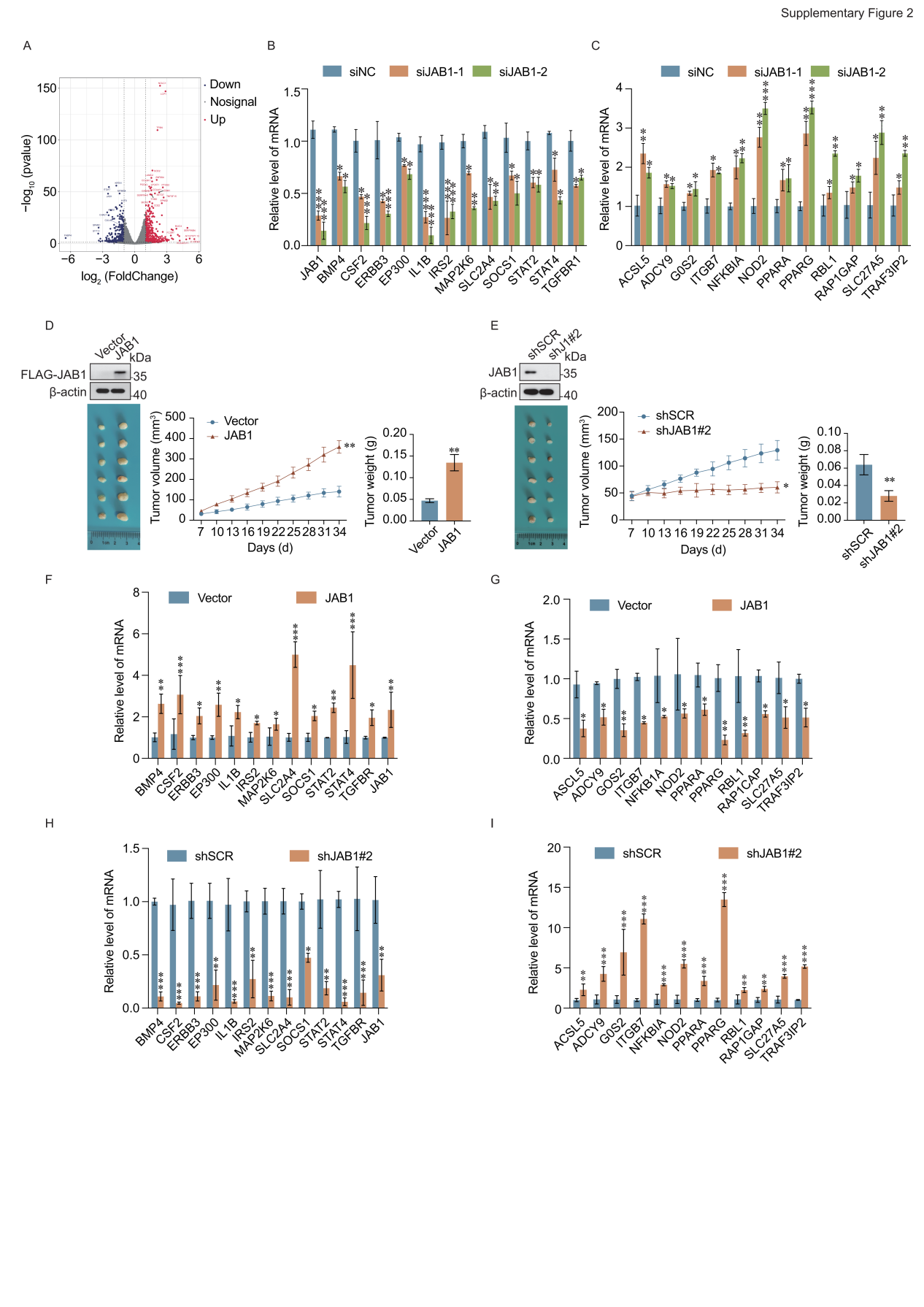


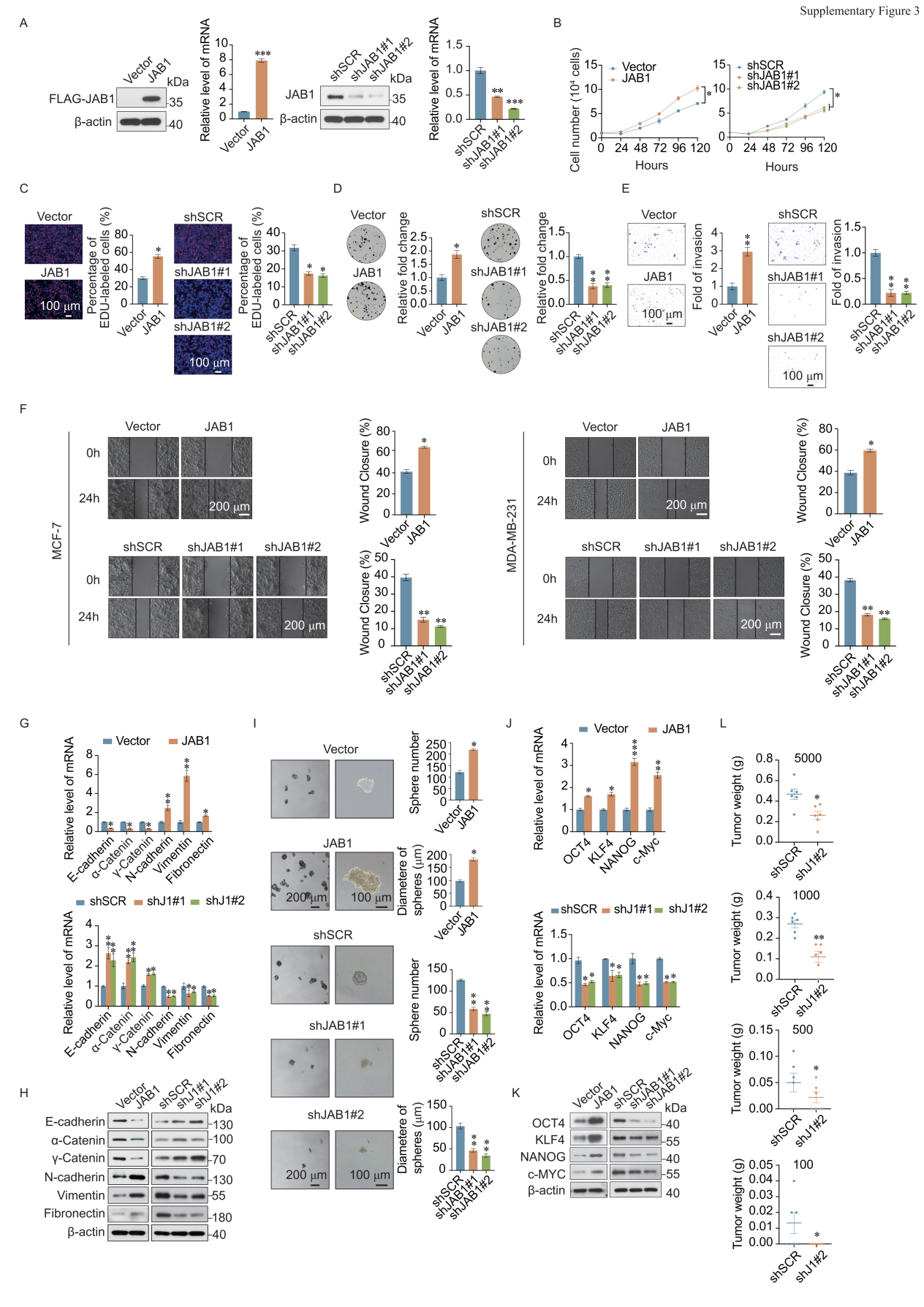


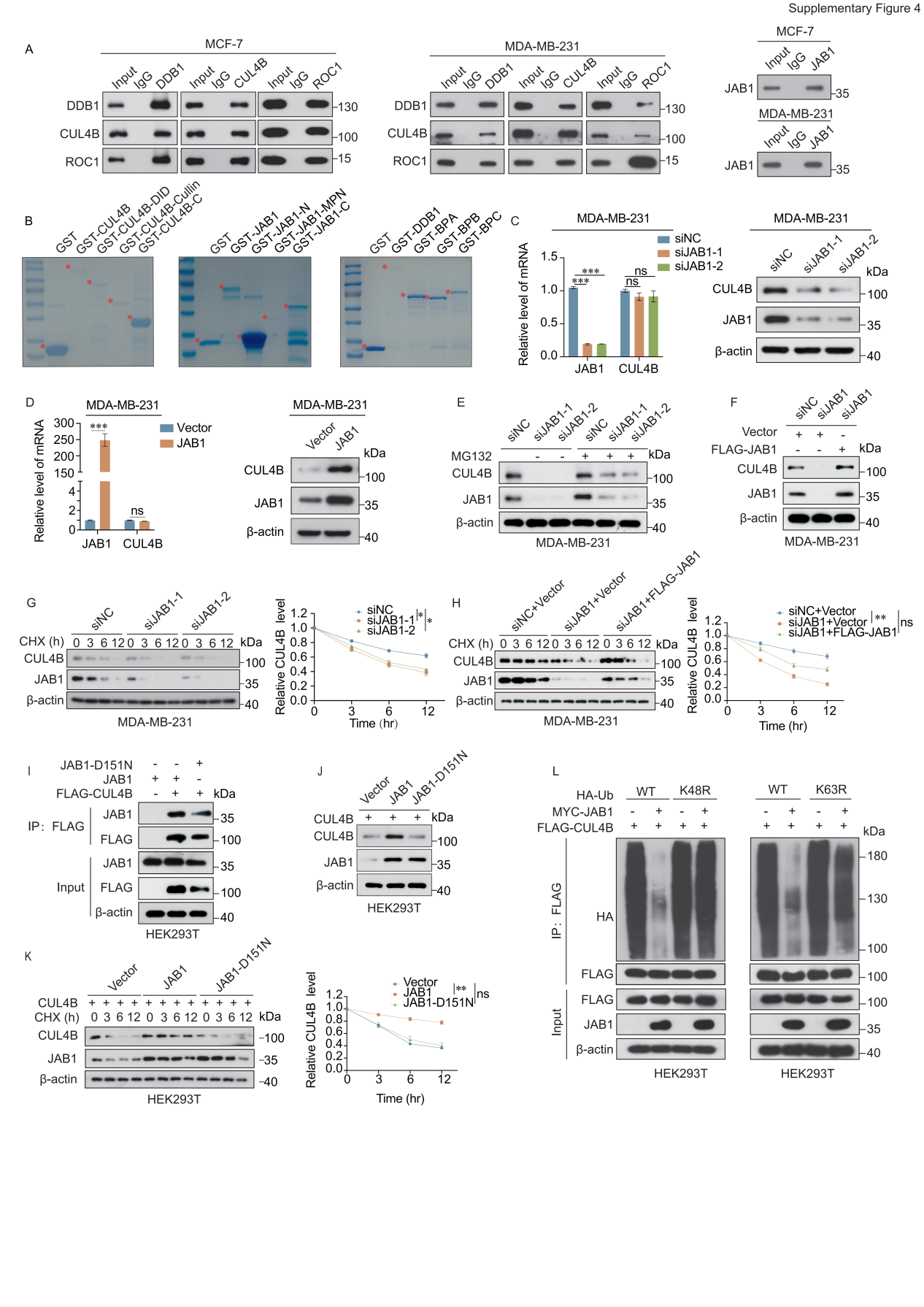


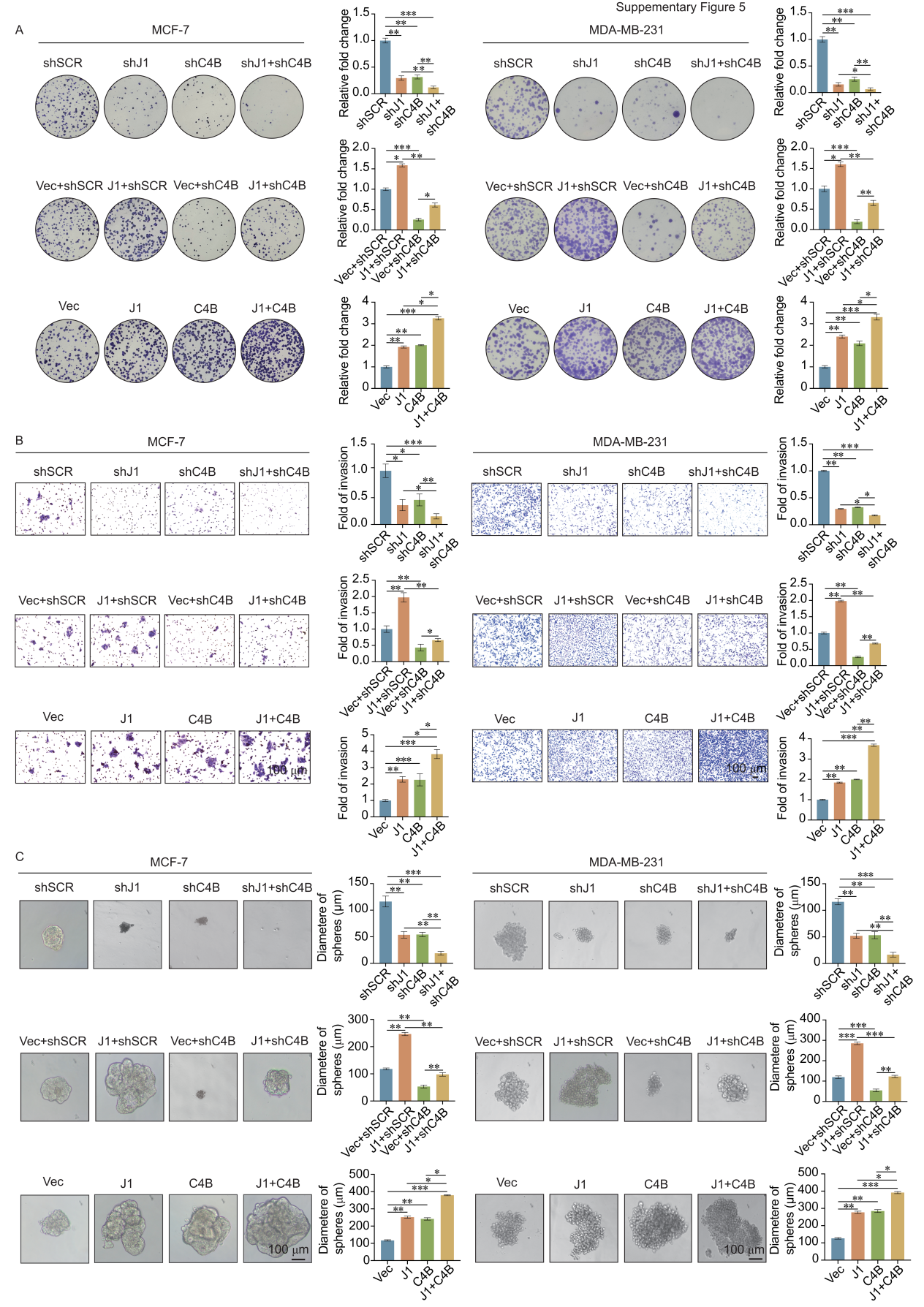


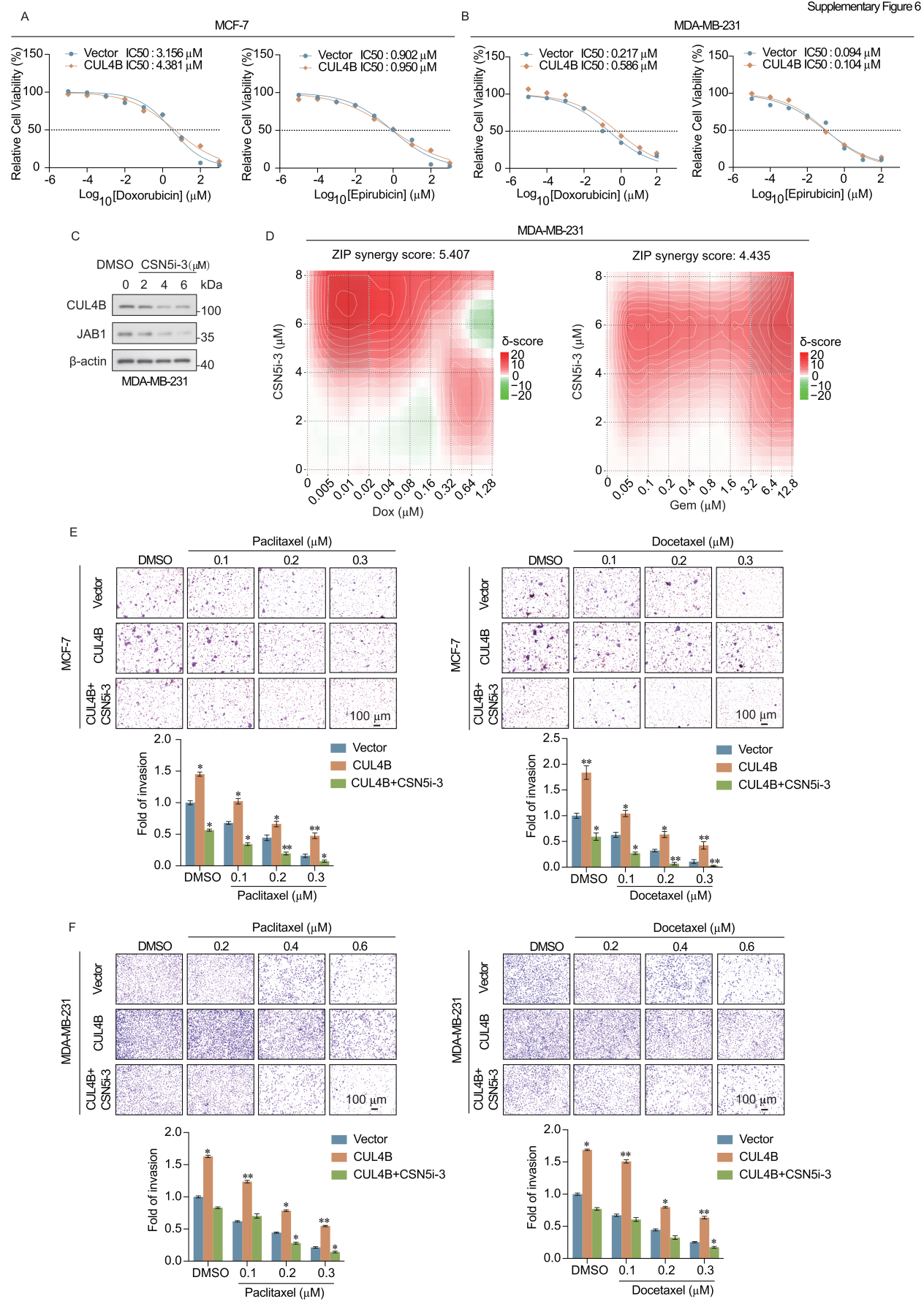


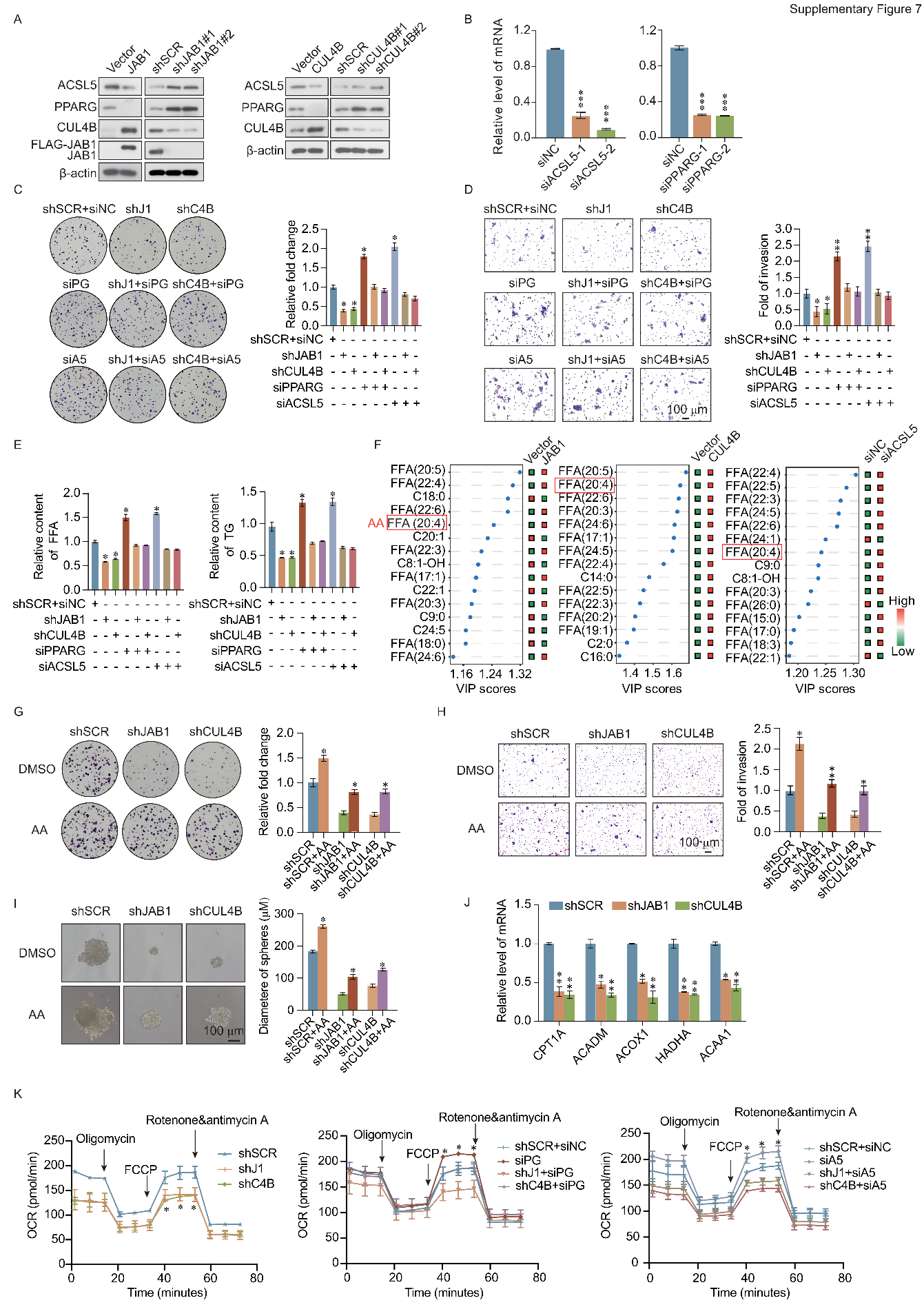

Supplement: Supplementary file 1 — Supplementary material file Figures [file 41418_2025_1642_MOESM1_ESM.docx]
