# Supplementary material for: JAB1/CRL4B complex represses PPARG/ACSL5 expression to promote breast tumorigenesis
Source: Cell Death Differ. 2025 Dec 12;33(6):1175–91. doi: 10.1038/s41418-025-01642-0 (PMC13247160; doi:10.1038/s41418-025-01642-0)
Supplement: Supplementary file 2 — Supplementary material file Legends [file 41418_2025_1642_MOESM2_ESM.docx]

**Supplementary Figure 1.** **JAB1 is upregulated in breast cancer and is associated with poor prognosis.** (A, B) COP9 signalosome (CSN) family expression in normal and breast cancer tissues based on public datasets GSE72653 and GSE20713. (C, D) Expression of JAB1 across molecular subtypes in breast cancer patients from public datasets TCGA BRCA and GSE65194. (E, F) Kaplan–Meier survival analysis of CSN expression in breast cancer using public datasets TCGA BRCA and GSE9893. For the TCGA dataset, the median expression value was used as the cutoff threshold for survival analysis, while the GSE9893 dataset employed the optimal cutoff value determined by maximizing survival differences between groups. Results are expressed as mean ± SD; Two-tailed unpaired *t*-test; **p* < 0.05, ***p* < 0.01, ****p* < 0.001, ns: not significant.

**Supplementary Figure 2. JAB1 promotes breast cancer progression.** (A) Expression of JAB1 in different molecular subtypes of breast cancer patients. (B, C) The expression levels of selected oncogenes and tumor suppressor genes in JAB1-knockdown MCF-7 cells were assessed by qRT-PCR. (D, E) The efficiency of stable overexpression or knockdown of JAB1 was assessed by western blotting. Subsequently, the MCF-7 cells were injected into NOD-SCID mice (*n* = 6), and the tumor volume and weight were evaluated; J1, JAB1. (F-I) Using qRT-PCR technology, the expression levels of the aforementioned tumor suppressor genes and oncogenes were detected in tumors with *JAB1* overexpression or knockdown. Results are expressed as mean ± SD; two-tailed unpaired *t*-test; **p* < 0.05, ***p* < 0.01, ****p* < 0.001, ns: not significant.

**Supplementary Figure 3.** **JAB1 participates in breast cancer progression.** (A) JAB1 knockdown and overexpression efficiency in MCF-7 cells assessed by qRT-PCR and western blotting. (B) Cell proliferation curves in MCF-7 cells transfected with shSCR, shJAB1, Vector, or FLAG-JAB1. (C) Representative images of EdU assays conducted on MCF-7 cells following transfection with the respective lentiviral Vectors; Scale bar, 100 μm. (D) Representative images of colony formation assays in MCF-7 cells transfected with the corresponding lentiviral Vectors. (E) Representative images of cell invasion assays performed with MCF-7 cells using Matrigel Transwell chambers. Each image corresponds to one microscopic field per group; scale bar, 100 μm. (F) Wound healing assays in MCF-7 and MDA-MB-231 cells with JAB1 knockdown or overexpression; scale bar = 200 μm. (G, H) Expression of epithelial and mesenchymal marker mRNA (G) and protein (H) in MCF-7 cells; J1, JAB1. (I) Representative images of mammosphere formation assays in MCF-7 cells show both the number (left panel, scale bar: 200 μm) and diameter (right panel, scale bar: 100 μm) of spheres after 15 days of culture. (J, K) Expression of stemness marker mRNA (J) and protein (K) in MCF-7 cells; J1, JAB1. (L) Tumor weight in NOD/SCID mice injected with different cell numbers of shSCR- or shJAB1-transfected MDA-MB-231 cells. (Results were presented as mean ± SEM). Error bars represent the mean ± standard deviation of three independent experiments; two-tailed unpaired *t*-test; **p* < 0.05, ***p* < 0.01, ****p* < 0.001.

**Supplementary Figure 4. JAB1 can enhance the stability of CUL4B protein.** (A) Interaction of the CRL4B complex in MCF-7 and MDA-MB-231 cells; JAB1 self-immunoprecipitation assay in MCF-7 cells and MDA-MB-231 cells. (B) GST fusion proteins purified from BL21 *Escherichia coli*. (C) CUL4B protein and mRNA levels in JAB1-knockdown MDA-MB-231 cells. (D) CUL4B protein and mRNA levels in JAB1-overexpression MDA-MB-231 cells. (E) CUL4B protein levels in JAB1-knockdown MDA-MB-231 cells treated with or without MG132 (10 μM). (F) CUL4B protein levels in JAB1-knockdown MDA-MB-231 cells transfected with a JAB1-overexpression plasmid. (G) CUL4B protein levels in JAB1-knockdown and control MDA-MB-231 cells treated with cycloheximide (CHX, 50 μg/mL). (H) CUL4B protein levels in JAB1-knockdown MDA-MB-231 cells transfected with JAB1-overexpressing plasmids and treated with cycloheximide (CHX, 50 μg/mL). (I, J) Co-immunoprecipitation (Co-IP) and western blot analysis of HEK293T cells transfected with FLAG-JAB1 or FLAG-JAB1 mutants. (K) CUL4B levels in 293T cells transfected with FLAG-JAB1 or FLAG-JAB1 mutants and subjected to a cycloheximide (CHX, 50 μg/mL) pulse-chase experiment. (L) Ubiquitination status of CUL4B in HEK293T cells co-transfected with FLAG-CUL4B, MYC-JAB1, and HA-tagged ubiquitin mutants (K48R and K63R: only the lysine residues at position K48 or K63 is mutated to arginine). Two-tailed unpaired *t*-test; **p* < 0.05, ***p* < 0.01, ****p* < 0.001, ns: not significant.

**Supplementary Figure 5.** **JAB1 and CUL4B promote breast cancer cell proliferation, invasion, and stemness.** (A) Representative images of colony formation assays in MCF-7 and MDA-MB-231 cells transfected with shSCR, shJAB1, shCUL4B, Vector, FLAG-JAB1, or FLAG-CUL4B. (B) Representative images of Transwell assays in MCF-7 and MDA-MB-231 cells transfected with the corresponding lentiviral Vectors; scale bar, 100 μm. (C) Representative images of the diameter of mammospheres; scale bar, 100 μm; Vec, Vector, J1, JAB1, C4B, CUL4B; error bars represent the mean ± SD of three independent experiments; two-tailed unpaired *t*-test; **p* < 0.05, ***p* < 0.01, ****p* < 0.001.

**Supplementary Figure 6.** **Combined JAB1 inhibitors and chemotherapeutic drugs synergistically reverse drug resistance in breast cancer cells.** (A, B) IC_50_ values of doxorubicin and epirubicin in MCF-7 and MDA-MB-231 cells transfected with lentiviral control Vectors or FLAG-CUL4B and treated with continuous doses of doxorubicin or epirubicin for 48 h. (C) Effect of CSN5i-3 on JAB1 and CUL4B protein levels. (D) Effect of combined CSN5i-3 and doxorubicin or gemcitabine on MDA-MB-231 cells assessed using the ZIP model in Synergy Finder 2.0; results are presented as a synergy score matrix; Dox, doxorubicin; Gem, gemcitabine. (E, F) MCF-7(E) and MDA-MB-231(F) cell invasion capacity following treatment with the lowest effective dose of CSN5i-3 and varying doses of paclitaxel or docetaxel; cells were transfected with lentiviral control Vectors or FLAG-CUL4B; error bars represent the mean ± SD of three independent experiments; two-tailed unpaired *t*-test; (E, F)two-way ANOVA;**p* < 0.05, ***p* < 0.01.

**Supplementary Figure 7.** **JAB1 and CUL4B regulate fatty acid metabolism by suppressing PPARG/ACSL5 expression, thereby promoting pro-tumor phenotypes in breast cancer cells.** (A) The protein expression levels of ACSL5 and PPARG were detected in MCF-7 cells with JAB1 knockdown or overexpression (left panel). The protein expression levels of ACSL5 and PPARG were also detected in MCF-7 cells with CUL4B knockdown or overexpression (right panel). (B) The efficiency of ACSL5 or PPARG knockdown in MCF-7 cells was confirmed using qRT-PCR. (C) Colony formation assays were conducted in MCF-7 cells after transfection with control, shJAB1, shCUL4B, siPPARG, or siACSL5. (D) Transwell assays were conducted on MCF-7 cells with control, shJAB1, shCUL4B, siPPARG, or siACSL5 transfection. (E) The relative levels of serum-free free fatty acids (FFA) and serum-free triglycerides (TG) were detected in MCF-7 cells transfected with control, shJAB1, shCUL4B, siPPARG, or siACSL5. (F) VIP scores and heatmap analysis of fatty acids with significant alterations in the JAB1 overexpression, CUL4B overexpression, and ACSL5 knockdown groups. (G) Colony formation assays were performed in MCF-7 cells (shSCR, shJAB1, and shCUL4B groups) following supplementation with 15 μM arachidonic acid. (H) Transwell assays were conducted in MCF-7 cells (shSCR, shJAB1, and shCUL4B groups) following supplementation with 15 μM arachidonic acid. (I) Sphere formation assays were performed in MCF-7 cells (shSCR, shJAB1, and shCUL4B groups) following supplementation with 15 μM arachidonic acid. (J) The expression levels of metabolites involved in fatty acid oxidation were detected by qRT-PCR in MCF-7 cells. (K) Mitochondrial oxygen consumption rate (OCR) was examined in MCF-7 cells transfected with control, shJAB1, shCUL4B, siPPARG, or siACSL5. J1, JAB1; C4B, CUL4B; PG, PPARG; A5, ACSL5. The error bars indicate the mean ± SD derived from three separate experiments; two-tailed unpaired *t*-test, **p* < 0.05, ***p* < 0.01, ****p* < 0.001.
