# Supplementary material for: JAB1/CRL4B complex represses PPARG/ACSL5 expression to promote breast tumorigenesis
Source: Cell Death Differ. 2025 Dec 12;33(6):1175–91. doi: 10.1038/s41418-025-01642-0 (PMC13247160; doi:10.1038/s41418-025-01642-0)
Supplement: Supplementary file 3 — Supplementary material file Tables [file 41418_2025_1642_MOESM3_ESM.docx]

**Supplementary Table S1.** Clinical traits of patients related to Figure 1D

| **Clinical Samples** | **Age（years）** | **TNM** | **ER**  **Status** | **PR**  **Status** | **HER2**  **Status** | **Ki-67**  **(%)** | **Grade** |
| --- | --- | --- | --- | --- | --- | --- | --- |
| Patient #1 | 59 | pT2N1M0 | - | - | 2+ | 60% | II |
| Patient #2 | 52 | pT2N0M0 | + | - | 3+ | 60% | III |
| Patient #3 | 41 | pT1N0M0 | - | + | 3+ | 30% | II |
| Patient #4 | 60 | pT1N0M0 | - | - | - | 80% | III |
| Patient #5 | 64 | pT1N0M0 | + | + | - | 15% | II |
| Patient #6 | 50 | pT2N1M0 | - | - | - | 10% | II |

**Supplementary Table S2.** Mass spectrometry analysis of JAB1-containing protein complex in MDA-MB-231 cells

| **Gene Symbol** | **Score** | **Coverage (%)** | **Peptides** |
| --- | --- | --- | --- |
| COPS5 | 21 | 58.08 | 41 |
| DDB1 | 9 | 11.11 | 13 |
| CUL4B | 14 | 9.111 | 2 |
| COPS4 | 12 | 51.97 | 53 |
| KIF11 | 19 | 26.52 | 53 |
| GPS1 | 12 | 41.84 | 37 |
| COPS3 | 19 | 40.43 | 31 |
| COPS2 | 10 | 50.11 | 28 |
| PRMT5 | 15 | 29.51 | 24 |
| COPS7A | 13 | 42.55 | 22 |
| PRSS1 | 16 | 14.94 | 17 |
| PRSS3 | 13 | 19.43 | 17 |
| COPS6 | 17 | 20.86 | 13 |
| PRSS3P2 | 13 | 19.43 | 12 |
| COPS8 | 12 | 23.92 | 11 |
| PRSS3P2 | 13 | 19.43 | 12 |
| KCTD5 | 12 | 32.48 | 10 |
| CUL4A | 20 | 21.61 | 8 |
| PPM1B | 13 | 17.12 | 7 |
| COPS7B | 20 | 29.92 | 7 |
| SRSF1 | 13 | 19.37 | 5 |
| DDX21 | 16 | 8.779 | 5 |
| EEF1A1 | 13 | 12.85 | 5 |
| HSPA8 | 20 | 21.61 | 4 |
| DDB2 | 13 | 17.12 | 4 |

**Supplementary Table S3.** The information for the plasmid

| **Plasmid** | **Expression** | **Tags** | **Promoter** |
| --- | --- | --- | --- |
| pCMV-tag2b-CUL4B | Mammalian | FLAG | CMV |
| pCMV-tag2b-DDB1 | Mammalian | FLAG | CMV |
| pcDNA3.1-JAB1 | Mammalian | FLAG | CMV |
| pcDNA3.1-JAB1-D151N | Mammalian | FLAG | CMV |
| pcDNA3.1-JAB1 | Mammalian | MYC | CMV |
| pCMV-HA-Ub | Mammalian | FLAG | CMV |
| pCMV-HA-Ub-K0 | Mammalian | FLAG | CMV |
| pCMV-HA-Ub-K48 | Mammalian | FLAG | CMV |
| pCMV-HA-Ub-K48R | Mammalian | FLAG | CMV |
| pCMV-HA-Ub-K63 | Mammalian | FLAG | CMV |
| pCMV-HA-Ub-K63R | Mammalian | FLAG | CMV |
| pGEX-4T-3-CUL4B-DID | Bacterial | GST | Tac |
| pGEX-4T-3-CUL4B-Cullin | Bacterial | GST | Tac |
| pGEX-4T-3-CUL4B-C | Bacterial | GST | Tac |
| pGEX-4T-3-DDB1-BPA | Bacterial | GST | Tac |
| pGEX-4T-3-BPB | Bacterial | GST | Tac |
| pGEX-4T-3-BPC | Bacterial | GST | Tac |
| pGEX-6P-1-N | Bacterial | GST | Tac |
| pGEX-6P-1-MPN | Bacterial | GST | Tac |
| pGEX-6P-1-C | Bacterial | GST | Tac |
| pGEX-6P-1-MPN-D151N | Bacterial | GST | Tac |

**Supplementary Table S4.** siRNA and shRNA sequences (5′→3′)

| siNC | UUCUCCGAACGUGUCACGUTT |
| --- | --- |
| siJAB1-1 | GCAGUUCCAGGAACCAUUUTT |
| siJAB1-2 | CCAGACUAUUCCACUUAAUTT |
| siJAB1-3 | GCUUGAGCUGUUGUGGAAUTT |
| siPPARG-1 | CCGUGGAUCUCUCCGUAAUTT |
| siPPARG-2 | GGUUGCAGAUUACAAGUAUTT |
| siACSL5-1 | GCUUGUUACACGUACUCUATT |
| siACSL5-2 | GCUGCUGCCUUUCUCAAAUTT |
| shSCR | TTCTCCGAACGTGTCACGT |
| shJAB1#1 | CCAGACTATTCCACTTAATAA |
| shJAB1#2 | CGCCTTTAGGACATACCCAAA |
| shCUL4B #1 | GGATTCATTGGATAGCGTTCT |

**Supplementary Table S5.** The primers used in quantitative real-time PCR (qPCR)

| **Gene** | **Strand** | **Sequence (**5′→3′**)** |
| --- | --- | --- |
| *GAPDH* | F | GTCTCCTCTGACTTCAACAGCG |
| *GAPDH* | R | ACCACCCTGTTGCTGTAGCCAA |
| *JAB1* | F | GTCTCCTCTGACTTCAACAGCG |
| *JAB1* | R | ACCACCCTGTTGCTGTAGCCAA |
| *BMP4* | F | CTGGTCTTGAGTATCCTGAGCG |
| *BMP4* | R | TCACCTCGTTCTCAGGGATGCT |
| *CSF2* | F | GGAGCATGTGAATGCCATCCAG |
| *CSF2* | R | CTGGAGGTCAAACATTTCTGAGAT |
| *ERBB3* | F | CTATGAGGCGATACTTGGAACGG |
| *ERBB3* | R | GCACAGTTCCAAAGACACCCGA |
| *EP300* | F | GATGACCCTTCCCAGCCTCAAA |
| *EP300* | R | GCCAGATGATCTCATGGTGAAGG |
| *IL1B* | F | CCACAGACCTTCCAGGAGAATG |
| *IL1B* | R | GTGCAGTTCAGTGATCGTACAGG |
| *IRS2* | F | CCTGCCCCCTGCCAACACCT |
| *IRS2* | R | TGTGACATCCTGGTGATAAAGCC |
| *MAP2K6* | F | GGCTACTTGGTGGACTCTGTTG |
| *MAP2K6* | R | CATCGTGATGCCCAGACTCCAA |
| *SLC2A4* | F | CCATCCTGATGACTGTGGCTCT |
| *SLC2A4* | R | GCCACGATGAACCAAGGAATGG |
| *SOCS1* | F | TTCGCCCTTAGCGTGAAGATGG |
| *SOCS1* | R | TAGTGCTCCAGCAGCTCGAAGA |
| *STAT2* | F | CAGGTCACAGAGTTGCTACAGC |
| *STAT2* | R | CGGTGAACTTGCTGCCAGTCTT |
| *STAT4* | F | CAGTGAAAGCCATCTCGGAGGA |
| *STAT4* | R | TGTAGTCTCGCAGGATGTCAGC |
| *TGFBR* | F | GACAACGTCAGGTTCTGGCTCA |
| *TGFBR* | R | CCGCCACTTTCCTCTCCAAACT |
| *ACSL5* | F | GCTTATGAGCCCACTCCTGATG |
| *ACSL5* | R | GGAAGAATCCAACTCTGGCTCC |
| *ADCY9* | F | CTCAAAACGGCTGCCAAGACGA |
| *ADCY9* | R | GAGAAGTCTGACTGTTGGTGAGC |
| *G0S2* | F | GCCTGATGGAGACTGTGTGCAG |
| *G0S2* | R | TCCTGCTGCTTGCCTTTCTCCT |
| *ITGB7* | F | ATCGAGGACAGTGCAACCACGT |
| *ITGB7* | R | TCAGCTCCTCTGAGAAGCCAAG |
| *NFKBIA* | F | TCCACTCCATCCTGAAGGCTAC |
| *NFKBIA* | R | CAAGGACACCAAAAGCTCCACG |
| *NOD2* | F | GCACTGATGCTGGCAAAGAACG |
| *NOD2* | R | CTTCAGTCCTTCTGCGAGAGAAC |
| *PPARA* | F | TCGGCGAGGATAGTTCTGGAAG |
| *PPARA* | R | GACCACAGGATAAGTCACCGAG |
| *PPARG* | F | AGCCTGCGAAAGCCTTTTGGTG |
| *PPARG* | R | GGCTTCACATTCAGCAAACCTGG |
| *RBL1* | F | CGAACTGACAGTGGGAGTCTTC |
| *RBL1* | R | TCTCTTAGCACTCCCTGCGGTA |
| *RAP1GAP* | F | TCAGGGAGTTTCTGCTCACCAAG |
| *RAP1GAP* | R | GCTCATCGTGAAGGTTGTCCAG |
| *SLC27A5* | F | GGAAGTCTACGGCTCCACAGAA |
| *SLC27A5* | R | GTCGAACTGCACCAGCTCAAAG |
| *TRAF3IP2* | F | GAGGATAGAATCCGAGGCATTGA |
| *TRAF3IP2* | R | GTAAGCCATGCTCATCCTCGTC |
| *AXIN1* | F | GTATGTGCAGGAGGTTATGCGG |
| *AXIN1* | R | CACCTTCCTCTGCGATCTTGTC |
| *BAX* | F | TCAGGATGCGTCCACCAAGAAG |
| *BAX* | R | TGTGTCCACGGCGGCAATCATC |
| *EFNA5* | F | CTCCAAATGGACCGCTGAAGTTC |
| *EFNA5* | R | GCTTTAGACAGGACCTTCTTCCA |
| *NR1H3* | F | TGGACACCTACATGCGTCGCAA |
| *NR1H3* | R | CAAGGATGTGGCATGAGCCTGT |
| *PERP* | F | CCAGATGCTTGTCTTCCTGAGAG |
| *PERP* | R | AGTGACAGCAGGGTTGGCATGA |
| *PMAIP1* | F | CTGGAAGTCGAGTGTGCTACTC |
| *PMAIP1* | R | TGAAGGAGTCCCCTCATGCAAG |
| *RELN* | F | GTCTACCTTCCACTCTCCACCA |
| *RELN* | R | GTCCAGCATCACAAATCCCTCG |
| *SOD2* | F | CTGGACAAACCTCAGCCCTAAC |
| *SOD2* | R | AACCTGAGCCTTGGACACCAAC |
| *WWC1* | F | GAACTGAGCCTTGGTAACAGCG |
| *WWC1* | R | TGTCTCCAGCCACTGACTCGTC |
| *ZFP36L1* | F | CCCTTTGAGGAAAACGGTGCCT |
| *ZFP36L1* | R | GCAAAAGCCGATGGTGTGGAAG |
| *E-cadherin* | F | CATTTCTTGGTCTACGCCTG |
| *E-cadherin* | R | GAGAGGAGTTGGGAAATGTG |
| *α-catennin* | F | AGCTGAAAGTTGTGGAAGAT |
| *α-catennin* | R | CCAACATCTTTCAATTCCTGTTG |
| *γ-catennin* | F | GGACAAGAACCCAGACTACC |
| *γ-catennin* | R | GTGGCATCCATGTCATCTCC |
| *N-cadherin* | F | CACTGCTCAGGACCCAGAT |
| *N-cadherin* | R | TAAGCCGAGTGATGGTCC |
| *Vimentin* | F | ATTGAGATTGCCACCTACAG |
| *Vimentin* | R | ATCCAGATTAGTTTCCCTCAG |
| *Fibronectin* | F | CCATCCATTGATTTAACCAACTT |
| *Fibronectin* | R | TACCAGGCAGGAGATTTGTTAA |
| *OCT4* | F | CTGGGTTGATCCTCGGACCT |
| *OCT4* | R | CCATCGGAGTTGCTCTCCA |
| *KLF4* | F | CATCTCAAGGCACACCTGCGAA |
| *KLF4* | R | TCGGTCGCATTTTTGGCACTGG |
| *C-Myc* | F | AAACTTGAACAGCTACGGAAC |
| *C-Myc* | R | ATTTGAGGCAGTTTACATTATGG |
| *NANOG* | F | TCTGGACACTGGCTGAATCCT |
| *NANOG* | R | CGCTGATTAGGCTCCAACCAT |

**Supplementary Table S6.** The primers used in qChIP Assays

| **Gene** | **Strand** | **Sequence (**5′→3′**)** |
| --- | --- | --- |
| *ACSL5* | F | CACAGATCAATGGGGCAGCA |
| *ACSL5* | R | AGGACTGCAAGGCATCATTACT |
| *AXIN1* | F | TGACTGCCCTCCGATGTACT |
| *AXIN1* | R | GAGTTGAGAAACATGGGCCG |
| *BAX* | F | TTAGTCATCTATAACGTCCTGC |
| *BAX* | R | CATCTCCCGATAAGTGCC |
| *NFKBIA* | F | AGCATAGTGAGCCGAAACCC |
| *NFKBIA* | R | GGCACCCAAATTCGAGGAGA |
| *PPARA* | F | TTGGGACTCAGGAGGGTGGAG |
| *PPARA* | R | GTACATCGGAGGGCAGTCAGG |
| *PPARG* | F | CAGGTCAGAGTACGGGTGC |
| *PPARG* | R | GACCATGACCCTGCCTCAG |
| *RELN* | F | CCACAACCGAGCAGCACA |
| *RELN* | R | GCAGCGACAGAGCCTCATCT |
| *SOD2* | F | AAGCCCAGCCCTTCCTGTT |
| *SOD2* | R | GGTGCTGAACCGTTTCCGT |
| *WWC1* | F | GCTCACTCTGGTGGGTCTTT |
| *WWC1* | R | AACTGGCTGTTCACTTCCCC |
| *GAPDH* | F | AGCCACATCGCTCAGACACC |
| *GAPDH* | R | CCCATACGACTGCAAAGACCC |
