# Supplementary material for: JAB1/CRL4B complex represses PPARG/ACSL5 expression to promote breast tumorigenesis
Source: Cell Death Differ. 2025 Dec 12;33(6):1175–91. doi: 10.1038/s41418-025-01642-0 (PMC13247160; doi:10.1038/s41418-025-01642-0)

Figure 1：

C：Cell panel

JAB1

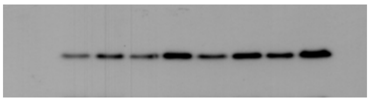

β-actin

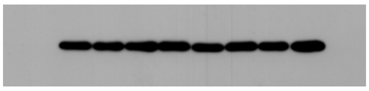

D：Tissue

JAB1

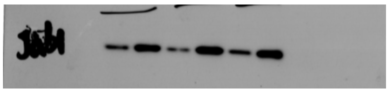

JAB1

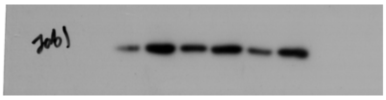

β-actin

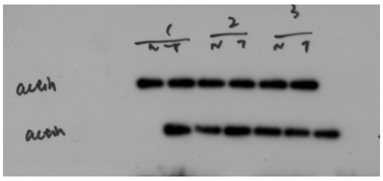

E：JAB1

β-actin

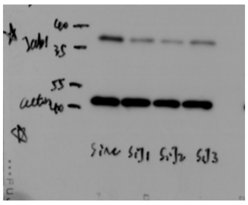

I： FLAG-JAB1

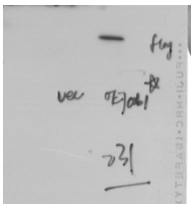

β-actin

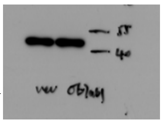

J：JAB1

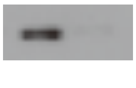

β-actin

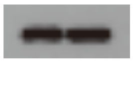

Supplementary Figure 2:

FLAG-JAB1 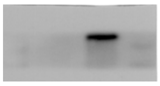

JAB1 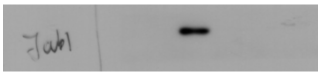

β-actin 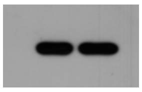

β-actin 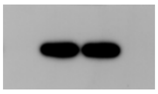

Figure 2：

A：MDA-MB-231

JAB1 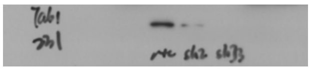

FLAG-JAB1 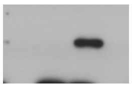

β-actin 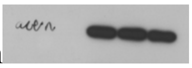

β-actin 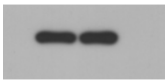

G：MDA-MB-231

E-Cadherin 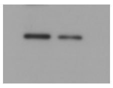 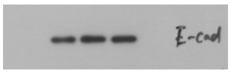

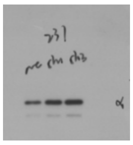

F-α-Catenin 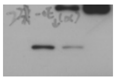

γ-Catenin 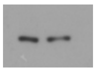 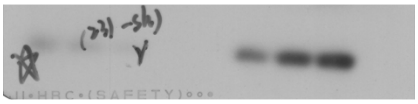

N-Cadherin 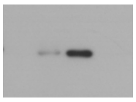 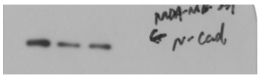

O-Vimentin 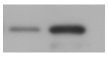 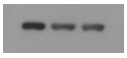

Fibronectin 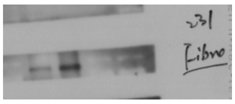 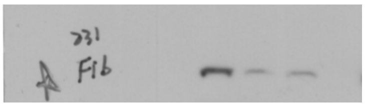

β-actin 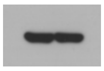 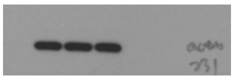

M：MDA-MB-231

OCT4

KLF4

NANOG

c-MYC

β-actin

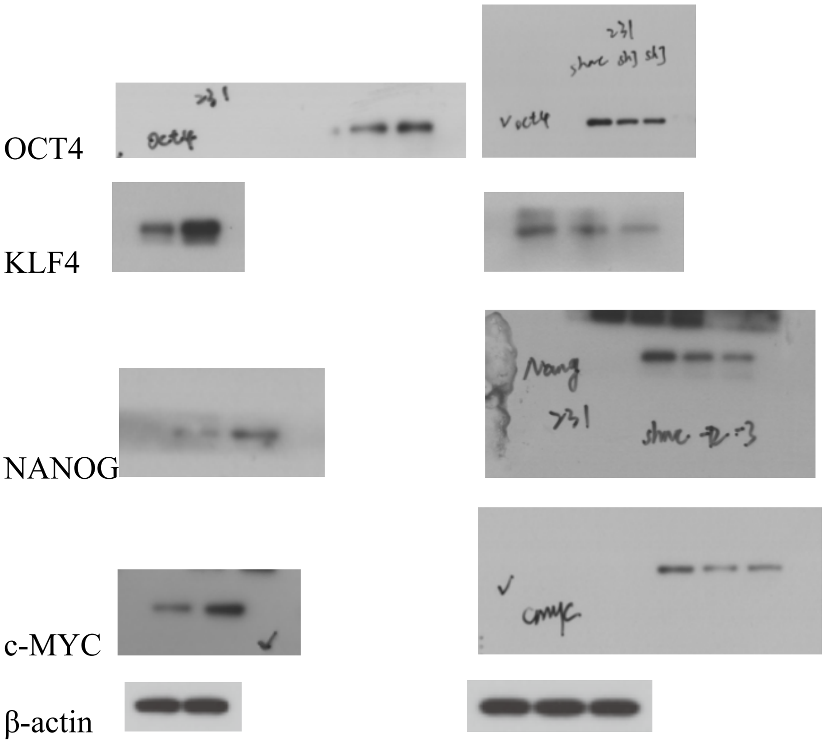

Supplementary Figure 3:

A：MCF-7

JAB1

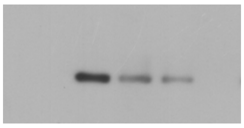

FLAG-JAB1

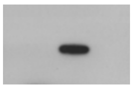

β-actin

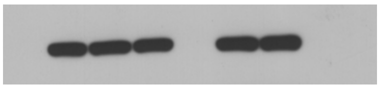

H：MCF-7

E-cadherin

F-α-Catenin

γ-Catenin

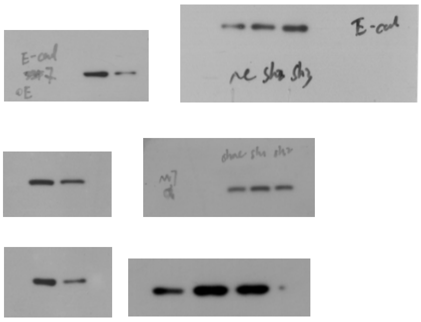

N-cadherin

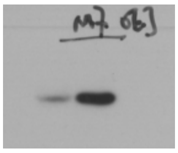

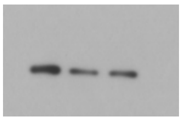

Vimentin

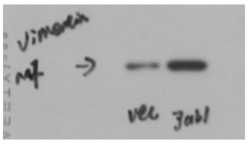

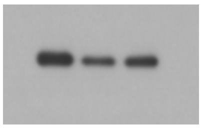

Fibronectin

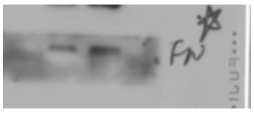

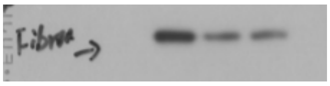

β-actin

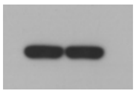

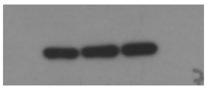

K：MCF-7

OCT4

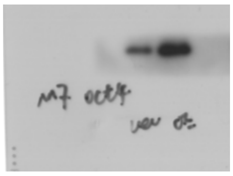

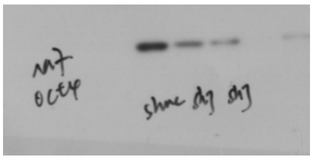

KLF4

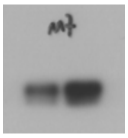

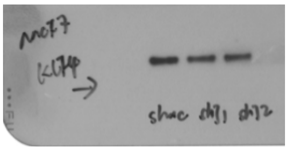

NANOG

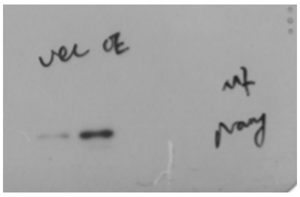

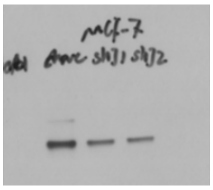

c-MYC

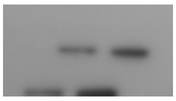

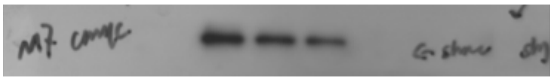

β-actin

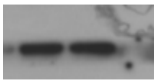

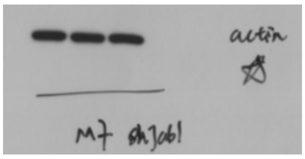

Figure 3

B：MDA-MB-231

JAB1 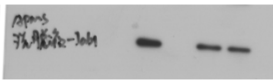 DDB1 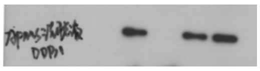

ROC1
CUL4B 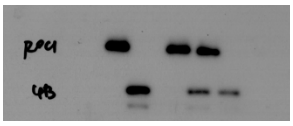

C：MCF-7

JAB1-DDB1

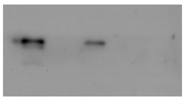 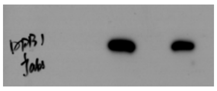

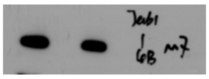 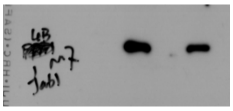

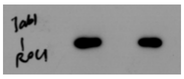 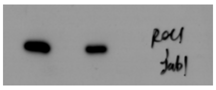

MDA-MB-231

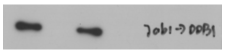 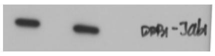

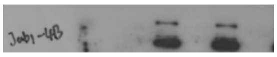 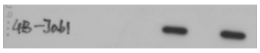

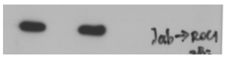 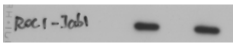

D：

GST-JAB1-DDB1 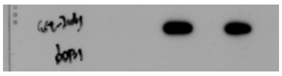 GST-DDB1-JAB1 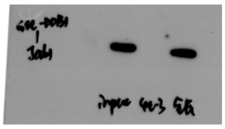

GST-JAB1-CUL4B 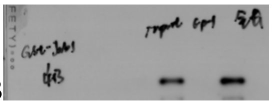 GST-CUL4B-JAB1 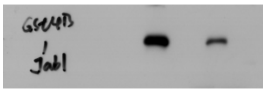

GST-JAB1-ROC1 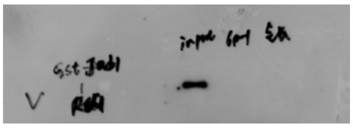 GST-ROC1-JAB1 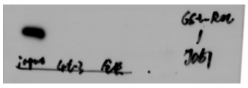

E：GST-CUL4B-JAB1

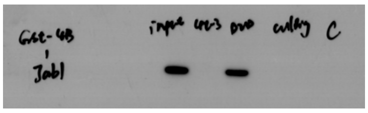

F：GST-DDB1-JAB1

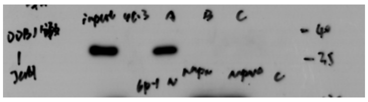

G：GST-JAB1-DDB1

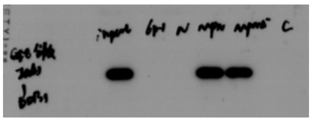

H：GST-JAB1-CUL4B

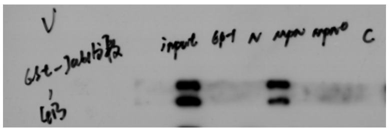

J：MCF-7

CUL4B

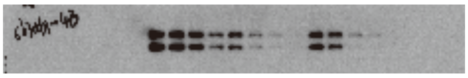

JAB1

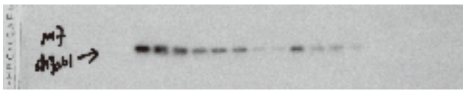

β-actin

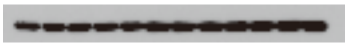

CUL4B

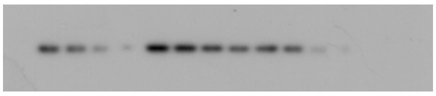

JAB1

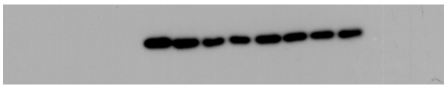

β-actin

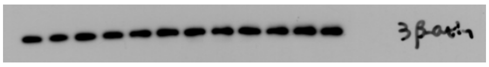

K：MDA-MB-231

CUL4B

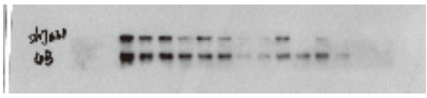

CUL4B

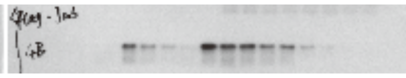

JAB1

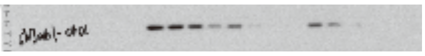

JAB1

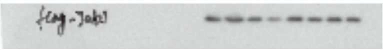

β-actin

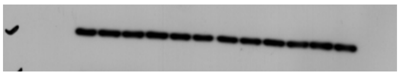

β-actin

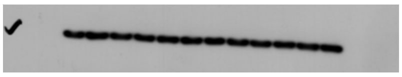

L： MDA-MB-231

IP：Ub 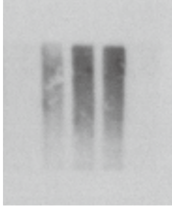

IP：CUL4B 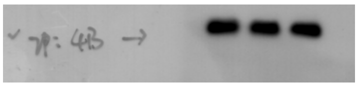

Input：JAB1 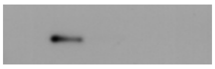

Input：CUL4B 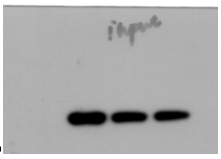

Input：β-actin 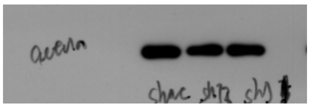

M： HEK293T

IP：HA 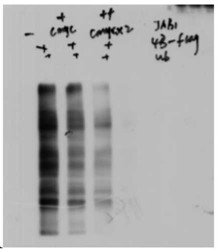

IP：FLAG 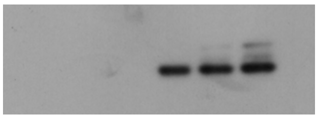

Input：FLAG 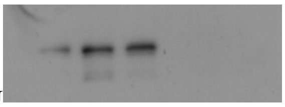

Input：JAB1 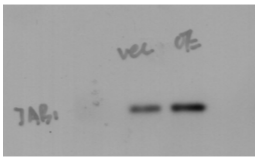

Input：β-actin 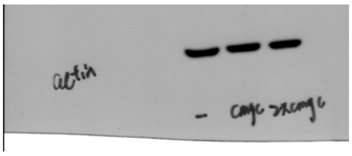

N：HEK293T

IP：HA 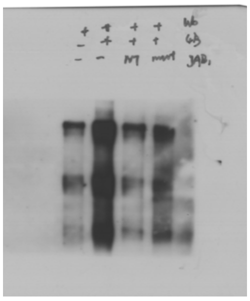

IP：FLAG 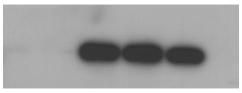

Input：FLAG 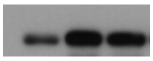

Input：JAB1 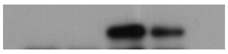

O：HEK293T

IP：HA 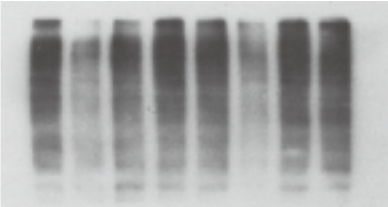

IP：FLAG 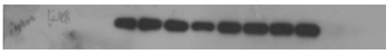

Input：FLAG 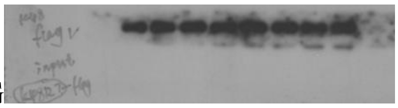

Input：JAB1 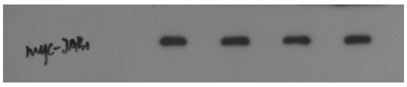

Input : β-actin

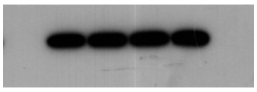

Input : β-actin

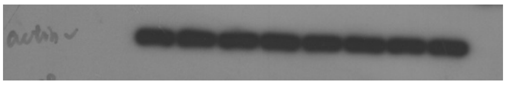

Supplementary Figure 4:

A：MCF-7

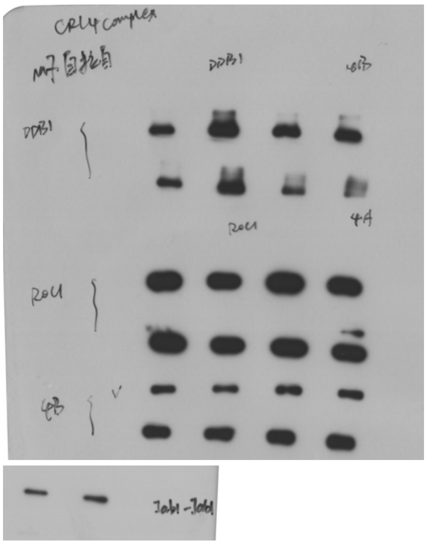

MDA-MB-231

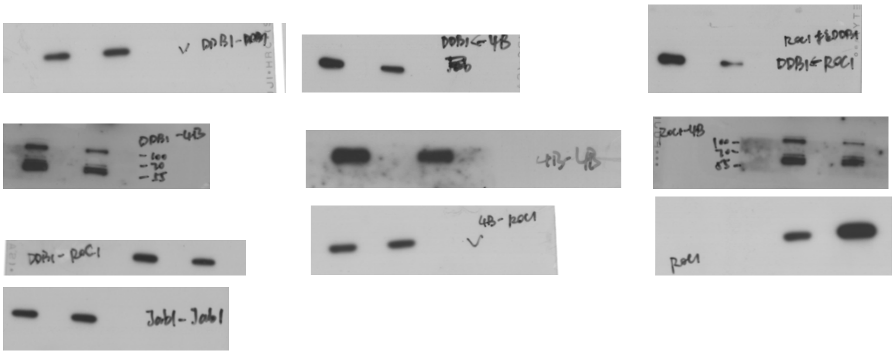

C：MDA-MB-231

D：MDA-MB-231

CUL4B

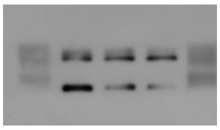

CUL4B

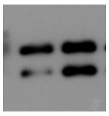

JAB1

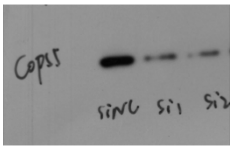

JAB1

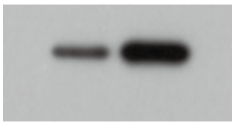

β-actin

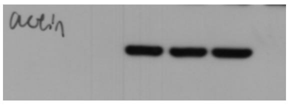

β-actin

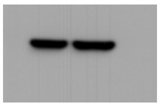

E：MDA-MB-231

CUL4B

JAB1

β-actin

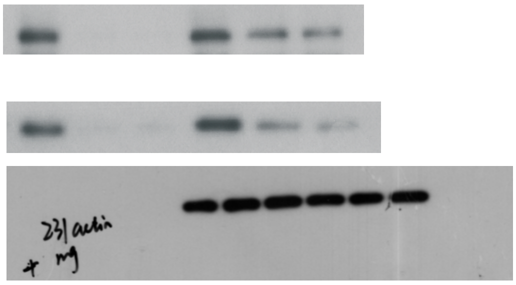

F：MDA-MB-231

CUL4B

JAB1

β-actin

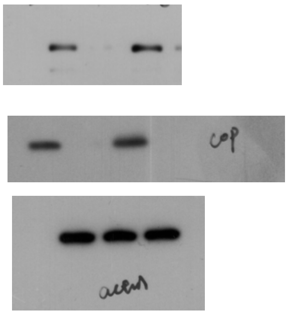

G：MDA-MB-231

CUL4B

JAB1

β-actin

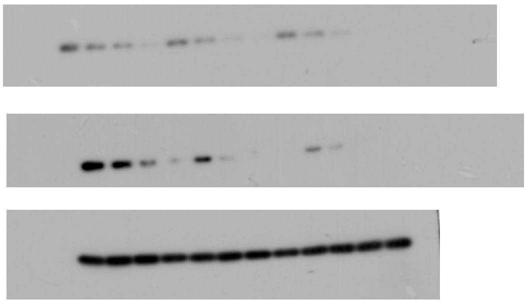

H：MDA-MB-231

CUL4B：

JAB1:

β-actin：

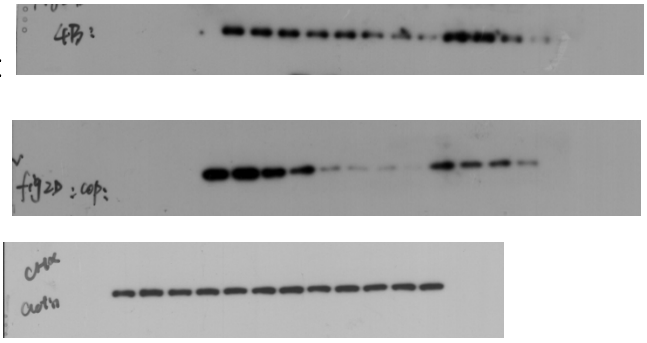

I： HEK293T

IP：FLAG JAB1

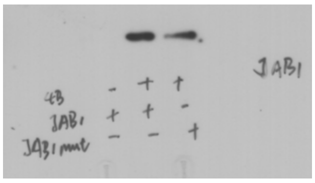

Input：FLAG

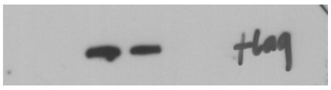

Input：JAB1

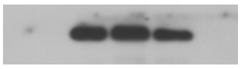

Input：FLAG

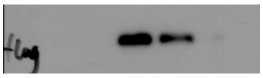

Input：β-actin

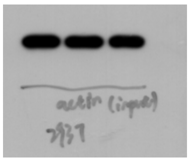

J：HEK293T

CUL4B

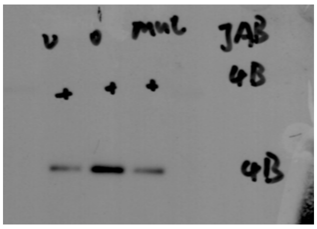

JAB1

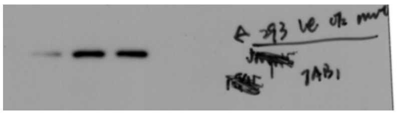

β-actin

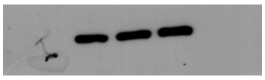

K：HEK293T

CUL4B

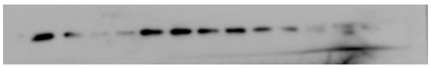

JAB1

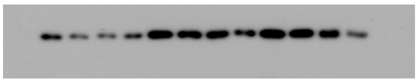

β-actin

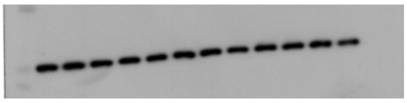

L：HEK293T

IP：HA

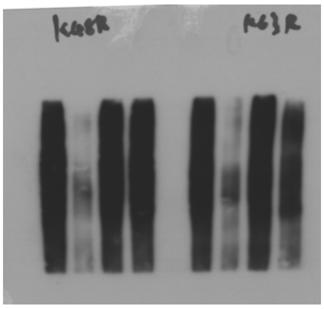

IP：FLAG

Input：FLAG

Input：JAB1

Input：β-actin

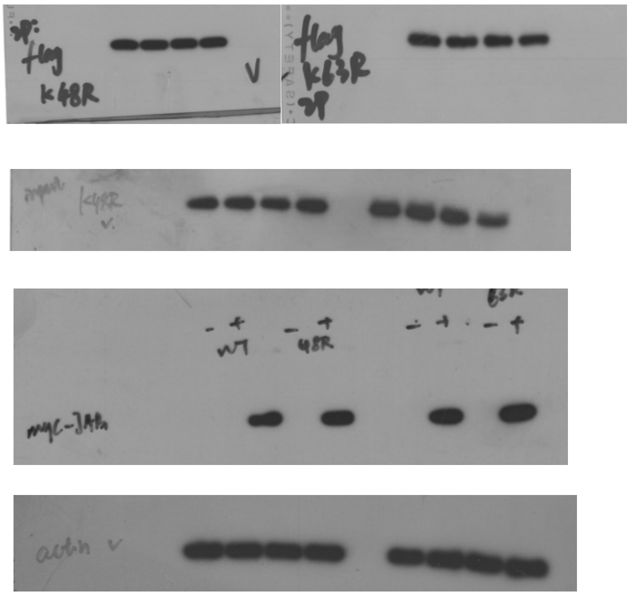

Figure 4：

E： MCF-7 MDA-MB-231

CUL4B

JAB1

E-cadherin

α-Catenin

γ-Catenin

N-cadherin

Vimentin

Fibronectin

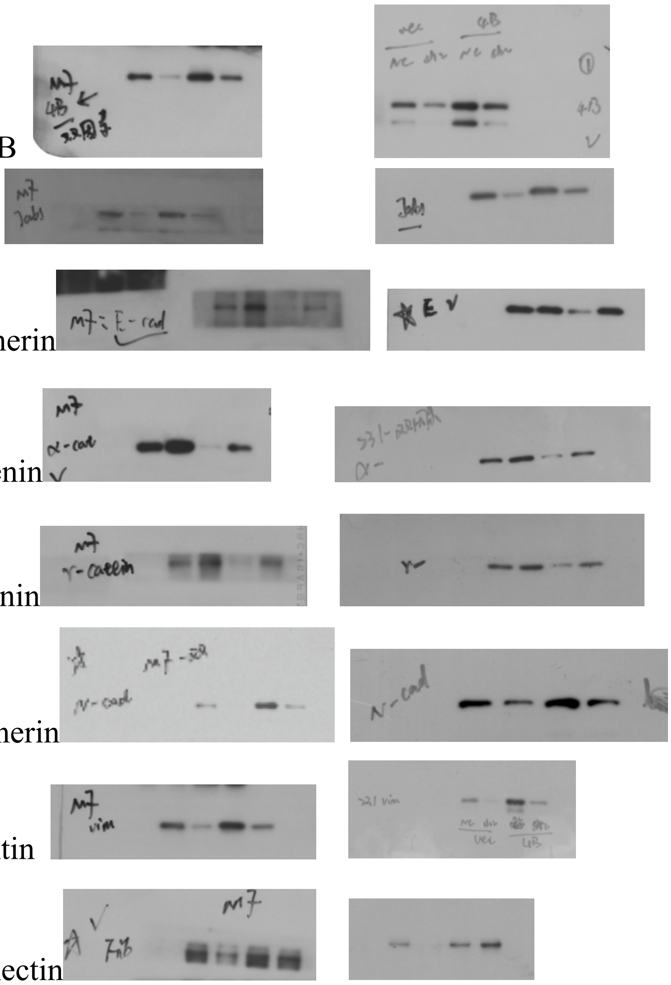

OCT4

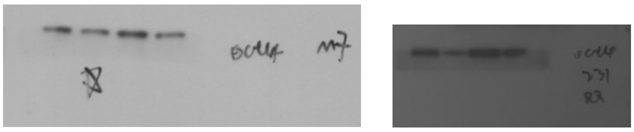

KLF4

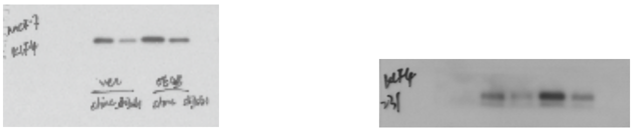

NANOG

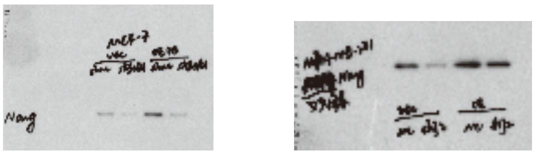

c-Myc

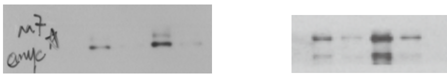

β-actin

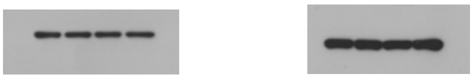

Supplementary Figure S6：

C：MDA-MB-231

CUL4B

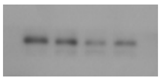

JAB1

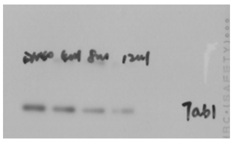

β-actin

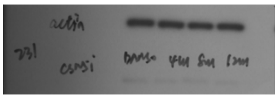

Figure 7：

A：MDA-MB-231

ACSL5

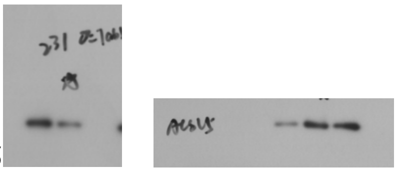

ACSL5

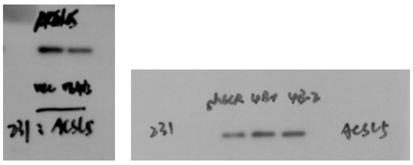

PPARG

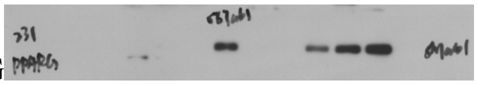

PPARG

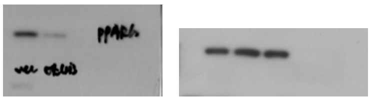

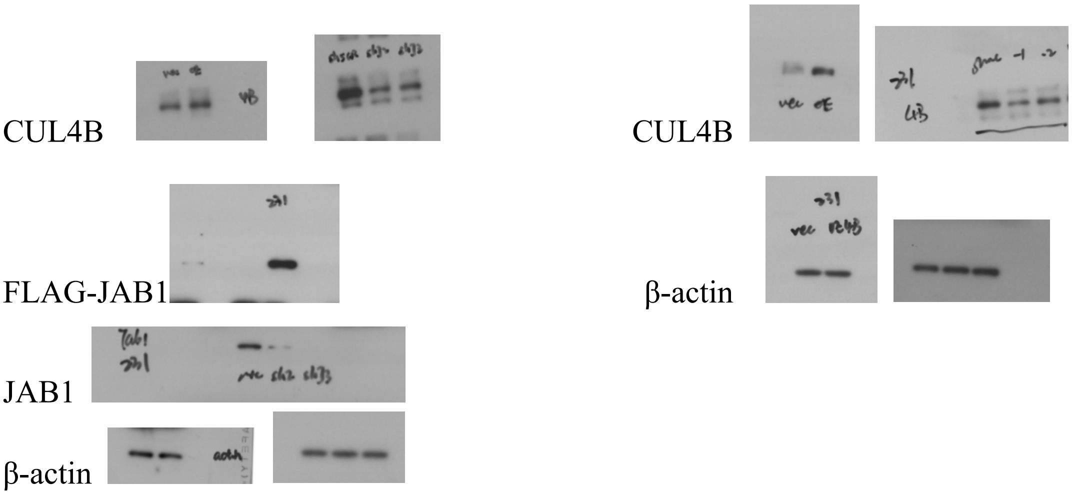

Supplementary Figure 7

A: MCF-7

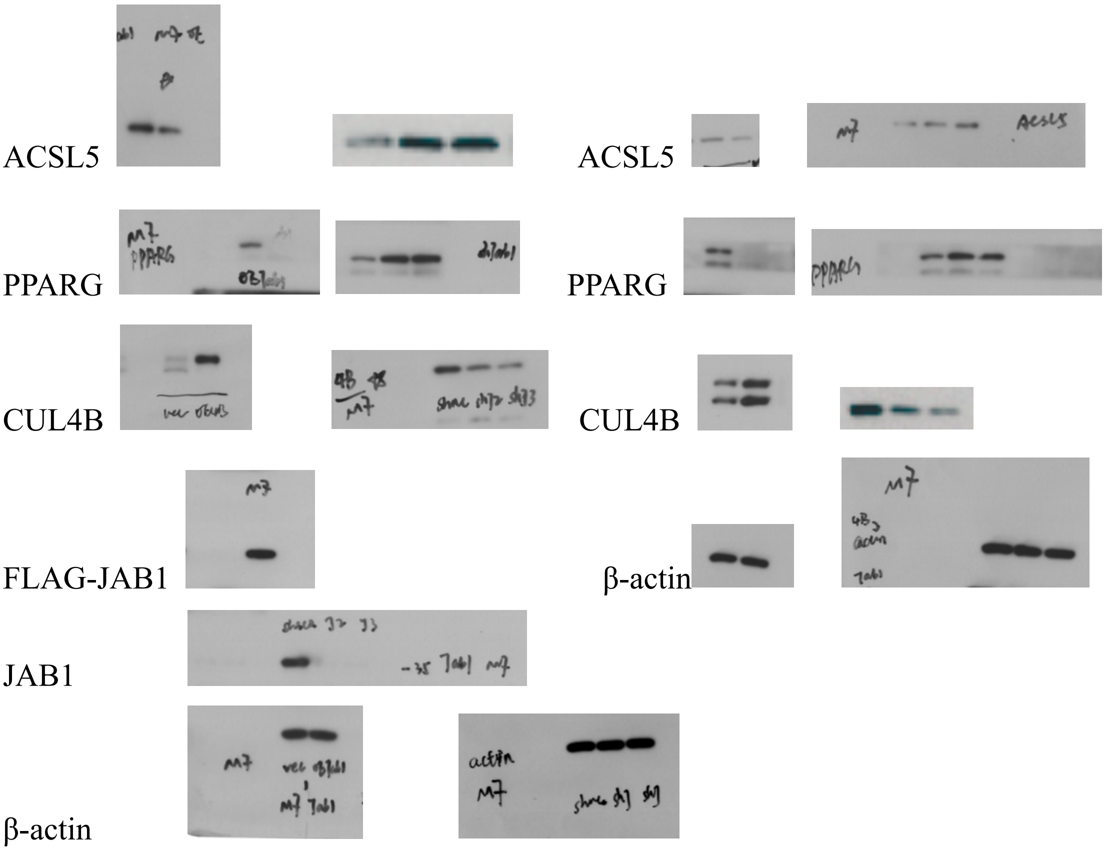

Supplement: Supplementary file 4 — Supplementary material-Uncropped Western Blots [file 41418_2025_1642_MOESM4_ESM.pdf]
