# Supplementary material for: JAB1/CRL4B complex represses PPARG/ACSL5 expression to promote breast tumorigenesis
Source: Cell Death Differ. 2025 Dec 12;33(6):1175–91. doi: 10.1038/s41418-025-01642-0 (PMC13247160; doi:10.1038/s41418-025-01642-0)
Supplement: Supplementary file 5 — Supplementary material- Raw qPCR Data [file 41418_2025_1642_MOESM5_ESM.pdf]

Figure 1C

	MCF-7			T47-D			SKBR3		
JAB1	3.162592	3.018551	3.10443	2.373731	2.452484	2.235074	6.885601	5.928554	6.683909
	BT474			MDA-MB-231			SUM159PT		
JAB1	3.784596	3.10975	3.871887	5.871747	5.139901	5.159516	4.273324	4.239498	3.740728
	BT549								
JAB1	7.066276	7.739247	7.921975						

Figure 1D

	Normal			Tumor		
#1	0.97990164	0.9254956	1.1026639	2.66353069	2.65318882	2.37485855
#2	1.13258711	0.97346952	0.90699737	2.59353747	2.39937307	2.34539816
#3	1.07556846	0.94832219	0.98040615	2.44053272	2.39586172	2.4406908
#4	1.01829493	0.99211337	0.98984027	3.43623597	3.42534563	3.39424515
#5	0.89912714	1.10372667	1.00766775	1.95799559	2.04874503	1.97097111
#6	0.88371323	0.83259783	1.35910612	3.29111498	3.2120335	3.1076627

Figure 1G

JAB1	siNC			siJAB1-1			siJAB1-2		
BMP4	0.9864	0.98825	1.02584	0.02686	0.02384	0.02381	0.01547	0.01676	0.01552
CSF2	0.90992	1.11085	0.90762	0.29617	0.30992	0.32196	0.5152	0.37901	0.51239
ERBB3	0.94025	1.01239	1.05054	0.30116	0.35751	0.45198	0.40716	0.38146	0.36691
EP300	1.18488	0.99412	0.94896	0.47886	0.42335	0.44951	0.52188	0.62142	0.5325
IL1B	0.98334	1.05916	0.96014	0.51559	0.51574	0.52653	0.17002	0.16303	0.16298
IRS2	0.97719	1.03677	0.98705	0.37842	0.38854	0.42826	0.40938	0.40273	0.39745
MAP2K6	0.90742	1.11703	0.98657	0.51802	0.40283	0.40943	0.48632	0.49262	0.55282
SLC2A4	1.00727	1.03975	0.95482	0.48373	0.48274	0.48254	0.60394	0.61384	0.66232
SOCS1	1.04804	0.99437	0.95956	0.52894	0.54892	0.52108	0.36735	0.38441	0.39959
STAT2	1.04064	1.01341	0.94823	0.33205	0.50114	0.5585	0.37804	0.38179	0.49611
STAT4	1.0112	0.98879	1.00013	0.4107	0.42534	0.41342	0.47581	0.46232	0.45295
TGFBR1	1.01927	0.9961	0.98494	0.46578	0.4815	0.50974	0.26448	0.25965	0.26558
	0.91005	0.98455	1.11608	0.6284	0.65206	0.46434	0.28339	0.27564	0.2673

Figure 1H

	siNC			siJAB1-1			siJAB1-2		
ACSL5	1.01544	1.04731	0.94882	2.14258	2.33631	2.25434	1.74394	1.63086	1.75515
ADCY9	0.98626	0.99945	1.01449	2.70259	2.72558	2.70103	3.44734	2.80215	3.26935
G0S2	0.86664	0.94874	1.21622	2.38888	2.41997	2.22131	2.56792	2.4779	2.46205
ITGB7	0.93284	1.01835	1.05267	2.42951	2.59147	2.68638	1.76893	1.8283	1.34102
NFKBIA	0.98334	1.02049	0.99652	1.53908	1.52008	1.52877	1.25163	1.22086	1.27611
NOD2	1.13921	0.73702	1.19101	2.52606	2.08799	2.28366	2.11177	2.46156	1.86769
PPARA	1.04709	0.9678	0.9868	3.49289	3.22867	3.24266	1.95374	1.98052	1.83625
PPARG	1.1372	1.08705	0.80894	2.13772	2.08654	2.16426	2.3702	2.48965	2.2105
RBL1	0.99042	0.99464	1.01512	1.55519	1.58074	1.59242	1.59453	1.60052	1.67238
RAP1GAP	1.13945	1.02764	0.85402	3.06223	2.58217	2.88702	2.0052	1.88263	1.83111
SLC27A5	1.19932	0.80216	0.83147	3.06786	3.00099	3.0427	2.29796	2.2427	2.46604
TRAF3IP2	0.98991	0.94162	1.07283	1.63197	2.18261	2.15461	1.7781	1.93592	2.0036

Figure 1K

	Vector					JAB1				
BMP4	1.00417	1.00178	1.00049	1.0056	1.00053	1.29678	1.69977	2.53727	1.4434	2.13159
CSF2	1.0112	1.00728	1.00021	1.0005	1.00205	1.4519	2.12083	2.91013	2.78393	2.38388
ERBB3	1.00003	1.05822	1.02884	1.03129	1.00073	2.24121	2.5138	1.82638	2.05155	2.75681
EP300	1.01559	1.02476	1.00224	1.00038	1.00002	1.15075	1.4605	3.13414	1.27643	1.97979
IL1B	1.00006	1.01085	1.00179	1.00024	1.00168	1.90931	1.47921	3.00289	1.12085	2.04624
IRS2	1.00196	1.02766	1.00483	1.00944	1.00488	2.03074	0.97575	1.6185	1.47997	1.77796
MAP2K6	1.02719	1.0077	1.00584	1.00107	1.00279	1.61685	1.69127	3.18384	1.1612	2.96077
SLC2A4	1.01025	1.00215	1.0421	1.00544	1.05243	2.43025	2.085	3.95241	1.4604	3.9541
SOCS1	1.02001	1.01703	1.01788	1.00144	1.00496	1.4336	1.27953	1.31228	1.60779	1.94194
STAT2	1.0047	1.01057	1.01824	1.00031	1.05241	2.44453	2.97292	2.45811	2.22372	3.53527
STAT4	1.00245	1.01277	1.00084	1.00079	1.00088	2.20863	1.35765	1.65625	1.87485	2.54806
TGFBR	1.28605	1.26519	1.00006	1.44295	1.01231	2.26516	2.25559	2.14362	2.56427	1.91629

Figure 1L

	Vector					JAB1				
ACSL5	1.00031	1.01487	1.06836	1.03358	1.06646	0.7298	0.41638	0.52816	0.47122	0.68585
ADCY9	1.00055	1.0003	0.73321	1.26471	1.03087	0.76647	0.33303	0.4698	0.62091	0.62853
G0S2	1.01452	1.00023	1.04129	1.00489	0.93479	0.40079	0.40615	0.47002	0.42404	0.46013
ITGB7	1.00137	1.00052	1.02941	1.00033	1.0409	0.6295	0.51732	0.57039	0.51236	0.47975
NFKBIA	1.00009	1.00095	1.15938	1.04795	0.96678	0.57638	0.86699	0.70863	0.55389	0.52938
NOD2	1.06315	1.0001	1.00512	1.00001	0.9876	0.44702	0.70206	0.58527	0.52854	0.66918
PPARA	1.02369	1.00534	0.93338	1.05254	0.77695	0.22124	0.13036	0.12825	0.15581	0.27685
PPARG	1.00563	1.00108	1.06452	1.00307	0.85577	0.40456	0.81578	0.67906	0.57426	0.5997
RBL1	1.00021	1.00022	0.94526	1.00978	1.10424	0.44215	0.43833	0.63925	0.62596	0.43086
RAP1GAP	1.00093	1.00428	1.13871	1.01381	0.91951	0.68856	0.5933	0.62995	0.51718	0.63286
SLC27A5	1.00481	1.00029	1.11345	1.02516	0.85541	0.64352	0.78194	0.68748	0.55809	0.6191
TRAF3IP2	1.00033	1.01869	0.89068	1.00097	1.19689	0.5661	0.36055	0.4933	0.50251	0.66955

Figure 1M

	shSCR					shJAB1#2				
BMP4	1.19315	1.05937	0.94396	1.2401	0.80639	0.4123			0.54879	0.60394
CSF2	1.00069	1.0131	1.00007	0.93885	1.01036	0.68156	0.56723	0.44322	0.49081	0.48036
ERBB3	1.00648	1.00847	1.00598	1.05428	1.00002	0.34457	0.5077	0.6026	0.43823	0.32332
EP300	1.00678	1.05195	1.0002	1.04559	1.00237	0.66223	0.86348	0.55706	0.51364	0.39434
IL1B	1.03465	1.15277	1.00001	1.00003	1.00001	0.36495	0.24765	0.42923	0.33784	0.36353
IRS2	1.00191	1.13766	1.01846	0.95397	1.00314			0.10646	0.5741	0.89115
MAP2K6	1.00134	1.03387	1.00011	0.98992	1.00002	0.15927	0.20807	0.26695	0.26869	0.27274
SLC2A4	1.0001	1.01169	1.00068	0.95976	1.00103	0.16304	0.1673	0.17707	0.1548	0.13239
SOCS1	1.00115	0.79741	1.1001	1.44124	1.03962	1.01133	0.70487	0.49196	0.55336	0.54238
STAT2	1.09498	1.19026	1.01535	1.01277	1.01645			0.18761	0.42312	0.82768
STAT4	1.05661	0.76781	1.0197	1.10937	1	1.18793	0.65986	0.26286	0.45898	0.64201
TGFBR	1.00096	1.00435	1.00126	0.85362	1.03928	0.23337	0.17901	0.3169	0.35289	0.56224

Figure 1N

	shSCR					shJAB1#2				
ACSL5	1.07329	0.93172	1.00007	0.96265	1.00209	7.74493	7.77318	6.93098	7.87545	8.1623
ADCY9	1.05757	0.94557	1.00012	1.00757	1	6.4355	5.38861	4.56049	3.51239	2.07004
G0S2	0.99195	1.00812	1.00118	0.94162	1.00259	5.0567	5.50092	4.08867	3.45537	2.63395
ITGB7	1.15969	0.8623	1.03637	1.12315	1.00206	2.00947	3.85362	3.48053	3.65825	3.20274
NFKBIA	0.83997	1.19051	1.00012	1.02054	1.00032	5.04316	5.69505	7.1764	5.23855	3.0308
NOD2	0.94368	1.05968	1.00006	0.97614	1.00181	4.27166	4.39781	5.12944	3.81758	2.57458
PPARA	0.7384	1.35428	1.00289	0.98556	1.00094	2.51107	2.19729	7.0302	4.01597	2.01954
PPARG	0.92618	1.0797	1.00373	0.98717	1.00154	5.84726	6.5206	3.68477	2.21756	
RBL1	1.42862	0.69998	1.00443	0.98457	1.00164	6.65518	3.08601	3.64133	3.48126	3.27256
RAP1GAP	0.97847	1.022	1.00133	0.97837	1.00002	8.49169	6.94668	6.86508	5.89722	4.55317
SLC27A5	0.89396	1.11862	1.0003	0.99833	1.00021	6.32733	6.26963	3.9435	3.12472	2.23819
TRAF3IP2	1.06256	0.94112	1.00048	0.96817	1.00057	4.15287	5.98922	3.86533	3.97421	3.75347

s-Figure 2C

	siNC			siJAB1-1			siJAB1-2		
JAB1	1.17555	1.13828	1.017	0.29662	0.32684	0.24093	0.23651	0.10958	0.08247
BMP4	1.10737	1.14076	1.08921	0.65214	0.63766	0.70874	0.50232	0.61842	0.57701
CSF2	0.90125	1.11729	0.99309	0.49084	0.45481	0.46116	0.16802	0.2885	0.19569
ERBB3	1.21419	0.93952	0.87661	0.43628	0.43932	0.4099	0.27675	0.31136	0.32912
EP300	1.08085	1.0163	1.017	0.76137	0.76376	0.77737	0.62851	0.70222	0.72196
IL1B	0.90292	1.04727	0.96055	0.29868	0.30921	0.20974	0.06561	0.0445	0.18816
IRS2	0.97701	1.06176	0.93088	0.32309	0.39229	0.08362	0.40425	0.27231	0.30425
MAP2K6	1.02337	1.05214	0.92873	0.67674	0.70547	0.7006	0.36857	0.36602	0.34389
SLC2A4	1.02574	1.09828	1.14605	0.53961	0.32988	0.53219	0.45481	0.37981	0.45481
SOCS1	1.19474	0.94606	0.95786	0.62851	0.71203	0.67638	0.53219	0.59874	0.37631
STAT2	0.90752	1.07177	1.02811	0.6552	0.55864	0.59874	0.55095	0.53589	0.66434
STAT4	1.09682	1.06683	1.07296	0.85461	0.66588	0.66128	0.47085	0.40707	0.42435
TGFBR1	1.04488	0.8909	1.07425	0.57038	0.56253	0.58642	0.64469	0.67674	0.63142

s-Figure 2D

	siNC			siJAB1-1			siJAB1-2		
ACSL5	1.32869	0.85857	0.87661	2.05623	2.46229	2.53151	1.95884	1.70527	1.91853
ADCY9	1.1487	0.78458	1.10957	1.65864	1.49485	1.55833	1.46409	1.50525	1.59107
G0S2	0.90125	1.00696	1.10191	1.30134	1.39474	1.31951	1.53688	1.56917	1.23971
ITGB7	0.80292	1.1355	1.09682	2.07053	1.73396	1.98736	1.85483	1.84272	1.83613
NFKBIA	0.93735	1.09936	0.97041	1.7411	2.31338	1.93746	2.12383	2.35653	2.21402
NOD2	1.05946	1.17555	0.80292	2.56685	2.67928	3.05252	3.67226	3.45017	3.37916
PPARA	0.9417	1.20025	0.88474	1.42076	1.63203	1.96791	2.11893	1.46747	1.5728
PPARG	1.13027	0.97041	0.91172	2.87185	2.55502	3.16748	3.34035	3.58278	3.65533
RBL1	0.73374	1.07425	1.26868	1.29534	1.52979	1.23399	2.4005	2.38165	2.27101
RAP1GAP	0.69096	1.05458	1.37237	1.32256	1.59475	1.54043	1.71713	1.97247	1.67018
SLC27A5	0.71698	1.01396	1.37554	1.77358	2.59668	2.35653	2.53737	3.10229	3.01746
TRAF3IP2	0.73374	1.07425	1.26868	1.42076	1.67791	1.35347	2.4005	2.3972	2.27101

Figure 1G

	Vector			JAB1		
BMP4	1.05049361	1.204935897	0.790028288	2.348177082	2.374839422	3.164945105
CSF2	1.934500489	0.470649934	1.09833078	4.053600842	2.228591131	2.934606848
ERBB3	1.056724327	0.886638505	1.067312772	1.773748298	1.878944691	2.485544745
EP300	0.955856079	0.949238706	1.102128048	1.939002036	2.973860209	2.830939863
IL1B	1.171511479	1.521411111	0.561056849	2.549168736	2.238651366	1.910417431
IRS2	1.034757475	1.24374196	0.777018111	1.804325576	1.65131877	1.653607071
MAP2K6	1.52624147	0.73651273	0.88960352	1.663913166	1.914189513	1.342615379
SLC2A4	0.997258925	0.830180791	1.207867757	5.162260339	4.314308006	5.514751367
SOCS1	1.15403169	1.094919835	0.7914071	2.173144394	2.17996998	1.77870252
STAT2	1.008113893	0.999966267	0.991984875	2.619055513	2.522182343	2.180169288
STAT4	0.835674512	0.865231747	1.383026168	4.782707591	5.92916298	2.762858712
TGFBR	0.949730221	1.073161694	0.981148129	2.034298618	2.292545019	1.548654874
JAB1	0.970092362	1.012954701	1.017646377	2.00751389	3.30833823	1.703579953

Figure 1H

	Vector			JAB1		
ASCL5	0.735334548	1.02715251	1.023975955	0.420257003	0.25797001	0.45110496
ADCY9	0.963457783	0.94274651	0.928589383	0.625975116	0.428785741	0.499603462
GOS2	1.126128252	0.887998323	0.98275481	0.380783353	0.263915947	0.418014851
ITGB7	1.067315516	1.026724563	0.9759611	0.437299308	0.462219382	0.44201852
NFKB1A	0.689701819	1.362656609	1.064025906	0.537789248	0.509454879	0.531641357
NOD2	0.840891042	1.574208731	0.755436487	0.498099748	0.597561565	0.598760106
PPARA	0.875109136	1.142714616	1.12471751	0.649415676	0.529541557	0.657015983
PPARG	1.200507131	0.884935354	0.941290575	0.262571743	0.271366351	0.161288322
RBL1	0.737181928	1.394374175	0.972850238	0.301464142	0.290743831	0.359070141
RAP1CAP	1.000736112	1.121178318	0.985703098	0.601158059	0.526351267	0.546349867
SLC27A5	1.239860769	0.916024587	0.880480924	0.670027435	0.443774691	0.426455879
TRAF3IP2	0.954770816	1.063645456	0.984700085	0.552066676	0.607403386	0.380836663

Figure 1I

	shSCR			shJAB1#2		
BMP4	1.024324814	0.963006798	1.01375487	0.073766352	0.097949729	0.156767132
CSF2	0.973176697	1.211760991	0.727858774	0.058325645	0.043130015	0.039819076
ERBB3	0.941538559	1.196678438	0.887532825	0.06106149	0.130783382	0.13886814
EP300	0.941538559	1.196678438	0.887532825	0.379764726	0.130783382	0.13886814
IL1B	0.746128927	0.934028329	1.234914664	0.082766739	0.050295973	0.058031279
IRS2	1.114561342	0.928909326	0.965878949	0.473273956	0.201760963	0.143090837
MAP2K6	0.936255501	0.933435513	1.144250992	0.158588091	0.067923134	0.115402683
SLC2A4	0.985535854	1.133289497	0.895337361	0.063257748	0.054760508	0.185468103
SOCS1	0.947520267	1.083534523	0.974021938	0.514464286	0.476838139	0.429470316
STAT2	1.334325931	0.885426492	0.846419309	0.258066251	0.144337437	0.157718653
STAT4	1.039334425	1.086783116	0.938484055	0.035265185	0.101110922	0.041495992
TGFBR	0.803054078	1.366717885	0.911121574	0.282257513	0.080435539	0.070981249
JAB1	1.022428269	1.232425283	0.793608941	0.478482829	0.195483544	0.254674112

Figure 1J

	shSCR			shJAB1#2		
ACSL5	1.238790205	0.941770601	0.85715055	1.783672224	3.11329081	1.959140382
ADCY9	1.732505746	0.799314148	0.722117411	5.322794497	3.673301228	3.784898195
G0S2	0.639771884	0.981978944	1.591741993	10.09763591	6.166297448	4.579342319
ITGB7	1.201330471	0.835787432	0.995959482	10.53072071	11.76172131	10.97362851
NFKBIA	1.814374107	0.681950066	0.808203235	3.03882124	2.90628268	2.795602774
NOD2	1.604194247	0.576719551	1.080882215	5.72610815	5.864220408	4.913453331
PPARA	1.187157225	0.840634521	1.002038803	3.914225574	2.770297785	3.477239089
PPARG	0.879897664	0.889392301	1.277834089	14.26337207	13.64376989	12.55221835
RBL1	1.73743205	0.729217061	0.789287728	1.892160185	2.419013748	2.426107417
RAP1GAP	0.872124592	0.83641732	1.370876873	2.057522833	2.686836328	2.446820792
SLC27A5	1.336769734	0.582902977	1.283355932	3.700616868	4.007961211	4.158487161
TRAF3IP2	1.027535605	0.973202287	1.075284283	5.38802358	5.193962166	4.948245882

Figure 2A left

	Vector			JAB1		
JAB1	0.92616	1.058	1.02054	2.59288	2.69179	2.55543

Figure 2A right

	shSCR			shJAB1#1			shJAB1#2		
JAB1	0.99205	1.00993	0.99811	0.25153	0.25245	0.22508	0.10098	0.10269	0.10559

Figure 2F

	Vector			JAB1		
E-cadherin	1.07158	1.10287	0.84616	0.33102	0.41545	0.52298
α-catenin	0.9924	1.07151	0.94042	0.18543	0.18807	0.15337
γ-catenin	1.07394	0.92428	1.00744	0.33859	0.35203	0.37462
N-cadherin	1.13669	0.89377	0.98431	1.95935	1.90442	1.73219
Vimentin	0.83513	0.88848	1.34772	1.7164	1.73708	1.65836
Fibronectin	0.83407	1.05448	1.13699	1.9388	1.73674	1.92653

	shSCR			shJAB1#1			shJAB1#2		
E-cadherin	0.89031	1.15246	0.97462	1.58529	1.62799	1.5672	1.62686	1.63465	1.43766
α-catenin	1.00269	1.00454	0.99281	1.56321	1.60773	1.55502	1.62264	1.68015	1.44475
γ-catenin	1.03423	1.16303	1.08011	1.64728	1.71885	1.7067	1.72679	1.75885	1.56369
N-cadherin	1.02733	1.02413	0.95046	0.5715	0.56253	0.57493	0.66172	0.54762	0.55519
Vimentin	0.95729	1.08893	0.95931	0.5657	0.45856	0.48661	0.30674	0.32529	0.28059
Fibronectin	0.98616	0.97852	1.03629	0.50898	0.5128	0.50957	0.54042	0.52039	0.52625

Figure 2L

	Vector			JAB1		
OCT4	0.930946	0.95517	1.124591	1.632558	1.799791	1.698237
KLF4	0.927014	1.010957	1.067041	2.972156	2.815763	3.01029
NANOG	0.837333	0.970117	1.231054	1.828574	1.772606	1.75144
c-Myc	0.954544	1.19224	0.8787	1.829415	2.030662	1.778894

	shSCR			shJAB1#1			shJAB1#2		
OCT4	1.093472	0.914518	1.070513	0.625372	0.676868	0.65425	0.735397	0.694624	0.602544
KLF4	0.976272	1.024305	0.911815	0.552943	0.555474	0.511294	0.598067	0.51633	0.58263
NANOG	1.028318	0.972462	0.964995	0.512241	0.481097	0.439542	0.567598	0.428204	0.46015
c-Myc	0.953539	1.013816	1.034433	0.541136	0.554536	0.507293	0.592983	0.504863	0.580376

s-Figure 3A left

	Vector			JAB1		
JAB1	1.0536	0.97423	0.97423	8.18051	7.76203	7.76203

s-Figure 3A right

	shSCR			shJAB1#1			shJAB1#2		
JAB1	0.99223	0.94293	1.06882	0.47392	0.46872	0.44889	0.22189	0.22661	0.21298

s-Figure 3G

	Vector			JAB1		
E-cadherin	0.97626	1.02483	0.99951	0.31236	0.37209	0.34053
α-catenin	0.98533	1.00727	1.00757	0.27561	0.27942	0.36667
γ-catenin	0.95812	1.00521	1.0383	0.27884	0.31858	0.28874
N-cadherin	0.93016	1.08099	0.99454	2.77383	2.2507	2.4389
Vimentin	1.01705	0.85705	1.14724	5.19969	6.26	6.13501
Fibronectin	0.96024	1.0299	1.01117	1.69579	1.58626	1.73667

	shSCR			shJAB1#1			shJAB1#2		
E-cadherin	0.96554	1.03568	1.00039	2.58945	2.37022	2.9314	2.08755	2.12283	2.65289
α-catenin	0.90766	1.16326	0.9471	2.24715	2.2832	2.10608	2.60375	2.17659	2.51373
γ-catenin	1.0276	0.97315	1.0674	1.52581	1.60388	1.60854	1.58481	1.58684	1.66504
N-cadherin	1.00133	0.99867	0.95879	0.49181	0.53754	0.4345	0.47247	0.52407	0.50724
Vimentin	0.86259	1.1593	0.97894	0.65512	0.78661	0.4583	0.7161	0.74807	0.67789
Fibronectin	0.96591	1.03529	0.9728	0.51233	0.53247	0.50273	0.51449	0.47838	0.57026

s-Figure 3J

	Vector			JAB1		
OCT4	0.93939	1.02435	1.03922	1.58134	1.61391	1.63686
KLF4	1.01869	1.01797	0.96433	1.62111	1.68928	1.78272
NANOG	0.94525	1.00133	1.05652	3.17262	2.98948	3.30293
c-Myc	0.9862	0.93094	1.08922	2.50428	2.71754	2.4686

	shSCR			shJAB1#1			shJAB1#2		
OCT4	0.99484	1.00519	0.88892	0.44972	0.49101	0.4521	0.55072	0.51425	0.5083
KLF4	0.99823	1.00177	0.98834	0.53918	0.61793	0.77158	0.64023	0.62216	0.72658
NANOG	0.92706	1.07868	1.00287	0.48996	0.50158	0.42462	0.52768	0.46679	0.48107
c-Myc	1.02129	0.97915	1.00178	0.50227	0.52574	0.51667	0.50578	0.5124	0.53072

s-Figure 4C

	siNC			siJAB1-1			siJAB1-2		
JAB1	1.02827	1.06171	1.05185	0.20134	0.19181	0.18176	0.19299	0.19402	0.196
CUL4B	1.00594	0.97416	1.02047	0.97398	0.86103	0.89549	0.82647	0.92533	0.99312

s-Figure 4D

	Vector			JAB1		
JAB1	0.96275	1.02768	1.01072	266.497	251.475	228.231
CUL4B	0.98207	0.9916	1.02689	0.89323	0.90869	0.88449

Figure 6C

	shSCR			shAB1#1			shJAB1#2		
ACSL5	1.01422	1.04137	0.94681	2.04238	2.16333	2.04454	1.76241	1.61098	1.72555
ADCY9	0.94857	1.01815	1.03542	4.11561	4.38874	4.34849	2.82286	2.89099	2.56993
AXIN1	0.87153	1.11495	1.02911	2.36474	2.78606	2.27083	2.469	2.27892	2.22914
BAX	1.00613	0.9963	0.9976	2.02191	1.99806	1.8592	1.30639	1.28778	1.2943
EFNA5	1.03169	0.97022	0.99903	2.42086	2.25885	2.37873	1.58818	1.56085	1.48411
NFKBIA	0.98334	1.02049	0.99652	1.53908	1.52008	1.52877	1.25163	1.22086	1.27611
NR1H3	0.99841	0.98073	1.02128	2.49902	2.37793	2.45941	2.38175	2.56849	2.24705
PPARA	1.04709	0.9678	0.9868	3.49289	3.22867	3.24266	1.95374	1.98052	1.83625
PPARG	1.1372	1.08705	0.80894	2.13772	2.08654	2.16426	2.3702	2.48965	2.2105
PERP	0.96584	1.00111	1.03421	1.82494	1.81778	1.88223	1.8549	1.91426	1.85814
PMAIP1	0.98325	0.97185	1.0465	1.48139	1.49162	1.53126	1.40103	1.60035	1.60314
RBL1	0.99042	0.99464	1.01512	1.55519	1.58074	1.59242	1.59453	1.60052	1.67238
RAP1GAP	1.13945	1.02764	0.95402	3.06223	2.58217	2.88702	2.0052	1.88263	1.83111
RELN	1.01633	0.86953	1.13157	1.54586	1.62489	1.50421	1.56561	1.69283	1.68954
SLC27A5	1.19932	0.80216	0.83147	3.06786	3.00099	3.0427	2.29796	2.2427	2.46604
SOD2	0.92541	0.9258	1.16721	3.6782	3.62704	3.69099	2.77805	2.62942	2.58572
WWC1	0.9494	1.02727	1.02534	3.05611	3.0537	2.9993	1.95164	1.93401	1.90042
ZFP36L1	0.98309	1.06143	0.95833	2.79325	3.11945	2.92905	2.66165	2.61487	2.85151

Figure 6D

	shSCR			shCUL4B#1			shCUL4B#2		
ACSL5	1.03023	0.98064	0.98982	2.36729	2.37204	2.46661	1.31077	1.22633	1.26909
ADCY9	0.87033	1.06603	1.07782	1.86775	1.9182	1.9661	2.10396	2.00545	2.19196
AXIN1	0.99604	1.01141	0.99265	3.1246	2.92707	2.93888	2.50041	3.45325	3.03994
BAX	1.00118	0.99563	1.0032	1.41916	1.40821	1.48713	2.21984	2.09148	2.14208
EFNA5	0.99934	0.77219	1.29586	1.87534	1.77952	1.92795	1.96511	2.11274	2.10318
NFKBIA	0.94374	1.05293	1.00635	1.36162	1.37002	1.40876	1.43652	1.38259	1.4359
NR1H3	0.94563	0.99176	1.06628	1.56711	1.54657	1.55373	3.02469	3.35068	2.95487
PPARA	0.98322	1.02396	0.99327	2.10258	1.96585	1.45475	2.13316	2.45408	2.22268
PPARG	1.01293	0.97322	1.0144	2.17752	2.21443	2.26522	2.71285	2.45833	2.56909
PERP	0.97091	1.02493	1.00491	1.63498	1.76481	1.78564	2.12751	2.10053	1.9771
PMAIP1	0.92795	0.93914	1.14748	1.69235	1.78997	1.87326	1.22328	1.13383	1.15081
RBL1	1.00432	0.96126	1.03583	1.25606	1.268	1.25917	1.95037	2.0597	1.97825
RAP1GAP	0.92323	1.00368	1.07918	1.69677	1.46197	2.13453	1.25717	1.27653	1.23757
RELN	1.05236	0.98287	0.9668	1.93622	1.76176	1.78149	1.88988	1.92336	1.76974
SLC27A5	1.01953	0.97875	1.00214	1.353	1.22302	1.26737	2.62812	1.54319	2.09499
SOD2	1.38322	0.89388	0.80878	1.44069	1.42494	1.38546	3.47842	3.2581	2.69295
WWC1	0.96658	1.04146	0.99339	1.44042	1.42829	1.4775	2.52836	2.40832	2.55485
ZFP36L1	1.07885	0.9697	0.95587	2.60045	2.30239	2.53177	2.81806	2.69406	2.42175

Figure 6E

	IgG			JAB1		
ACSL5	1.06420895	0.79618187	1.18021413	2.45985483	2.59793918	2.24717196
AXIN1	0.83702506	1.287234	0.9281198	1.20773657	1.26852536	1.51353764
BAX	0.84535547	1.07051245	1.10501687	3.28435743	3.1186162	3.15827435
NFKBIA	1.28172005	0.98380583	0.79304427	4.78567882	3.51176449	4.33467038
PPARA	0.95299133	0.8718889	1.20351055	3.1486194	3.11470177	3.34916127
PPARG	1.39011338	0.7247354	0.99259092	3.08217558	3.51977007	2.65418675
RELN	1.07828835	1.17342498	0.79033235	4.28720078	2.64079743	2.77264972
SOD2	1.15158957	1.26528491	0.68629992	2.4543119	2.32991575	2.45294762
WWC1	0.86286493	1.09826866	1.05523354	1.41624114	1.42086495	1.43174249
GAPDH	1.13833806	0.76689503	1.145494	0.95207874	1.17194592	1.07365485

	IgG			CUL4B		
ACSL5	1.02738166	1.82717755	0.53270582	8.83107418	6.85005125	7.83652857
AXIN1	0.96264557	1.04704326	0.99213086	1.98207078	1.86492532	1.89354957
BAX	1.11409654	0.89758828	1.03864263	7.54802282	6.73859177	7.23746514
NFKBIA	0.88839796	1.03276485	1.12562167	4.33857294	4.75505847	3.16940125
PPARA	0.98811332	0.96221406	1.05177186	3.95058303	3.92535139	3.5661707
PPARG	1.43593818	0.89364852	0.69640881	11.6160357	12.3410309	11.97364595
RELN	0.90708591	1.0836455	1.10243141	2.47245367	2.87387608	2.6628495
SOD2	1.0725485	0.81488697	1.22716406	2.0716495	1.91253965	2.1143043
WWC1	0.87234895	0.97577812	1.17478579	2.63789308	3.30455824	2.36524523
GAPDH	1.17590148	1.12725809	0.75440699	0.93054842	0.74557344	0.71593434

	IgG			H2AK119ub1		
ACSL5	1.13888499	0.7869743	1.1157312	5.72792658	5.8228642	5.73764756
AXIN1	1.00227655	0.9736643	1.02471521	1.78376393	2.31079806	2.14261533
BAX	0.95619102	1.06495115	0.98203203	4.69995292	4.48284533	4.25141589
NFKBIA	0.7958216	1.14725714	1.09527584	2.89229582	3.18117022	2.34058259
PPARA	1.27915033	1.00764377	0.77583861	2.19449871	2.63828487	2.60801997
PPARG	1.050418	0.96752593	0.98395499	2.55098741	3.67126832	3.22061929
RELN	0.81831868	0.94967135	1.2867797	4.18048433	3.58003282	3.87686492
SOD2	1.15573513	1.38098557	1.28375659	1.9616671	1.91306312	2.23951016
WWC1	0.69712457	0.920669	1.55806687	3.2200099	4.36302872	2.80481107
GAPDH	0.90293828	1.6727721	0.6620719	0.91666223	1.23123654	1.30199779

Figure 6F

JAB1 ChIP

	shSCR			shJAB1			shCUL4B		
ACSL5	5.272629	5.29204	5.237492	0.096207	0.127023	0.137648	0.86287	0.804335	0.494241
AXIN1	5.482213	5.327496	5.326536	2.762549	2.430989	3.115074	2.330177	2.517288	2.397259
BAX	2.755817	2.544515	2.673626	1.609012	0.601374	0.602191	0.57265	0.463627	0.671494
NFKBIA	4.701012	4.657548	4.627827	1.265856	1.316084	1.214359	2.557326	2.281705	2.37365
PPARA	1.62787	1.214968	2.010908	0.635468	0.414936	0.988005	0.530515	0.411396	0.460252
PPARG	2.245269	2.607563	2.132589	0.673655	0.929331	0.480106	1.084759	1.154636	0.705974
RELN	3.049059	1.928146	2.335694	0.27564	0.260159	0.34109	0.876031	1.241041	0.405888
SOD2	3.016483	3.008006	3.040243	0.541744	0.725095	0.76371	1.047842	0.761688	0.497859
WWC1	2.301232	3.49054	2.994158	2.027472	2.073347	2.080561	1.525155	1.365424	1.822852
GAPDH	1.073977	0.727531	0.81852	0.79275	0.852139	0.754416	0.86287	0.804335	0.494241

CUL4B ChIP

	shSCR			shJAB1			shCUL4B		
ACSL5	17.16476	16.93178	17.03766	7.182391	6.131242	6.611337	4.005375	4.548271	5.05647
AXIN1	7.462703	9.433375	6.695549	5.376783	4.086834	4.008721	3.899741	4.057634	3.972619
BAX	11.42031	10.48151	9.168978	6.139015	5.952756	5.5629	3.513068	3.501842	3.465077
NFKBIA	13.80848	17.27121	15.28489	8.387303	8.944066	8.526486	2.642826	1.687797	2.086821
PPARA	13.18628	13.52864	13.38563	3.787373	6.663094	3.51137	3.636913	3.326597	2.934393
PPARG	11.97629	10.20394	13.27208	2.371986	2.038597	2.497384	2.608629	1.814258	2.516382
RELN	19.73018	16.75355	17.94763	6.052577	5.262354	8.72254	5.74651	3.127203	4.582648
SOD2	14.72301	11.41062	11.08015	3.050986	1.80293	2.169454	1.350951	1.194957	0.933024
WWC1	9.316539	7.01472	8.823485	3.514561	2.592369	2.999516	3.323017	3.743298	3.185087
GAPDH	0.920146	0.794605	0.752065	0.684865	0.858225	0.626095	0.726138	0.787268	0.213247

H2AK119ub1 ChIP

	shSCR			shJAB1			shCUL4B		
ACSL5	3.119376	3.480815	2.941883	2.898805	2.993757	3.056536	0.233866	0.239191	0.221399
AXIN1	4.135353	4.226962	4.103591	3.793998	3.299116	3.45813	2.43175	2.471085	2.500445
BAX	6.68521	6.943524	6.726385	6.327105	6.219097	6.654751	1.328083	1.436057	1.244346
NFKBIA	3.666727	3.723619	3.830935	2.06612	2.486208	2.868794	0.75312	0.811472	0.704268
PPARA	2.020523	2.115026	2.643205	2.668591	1.761672	1.909035	0.10358	0.462907	0.118902
PPARG	2.560437	2.602948	2.6154	2.674303	2.106637	2.397295	0.305946	0.202638	0.265083
RELN	3.41531	3.372126	3.514144	3.964439	3.72469	3.425772	0.706065	0.969814	0.79901
SOD2	4.12863	4.025559	4.33132	2.967288	3.861055	4.702471	0.416385	1.011462	0.669315
WWC1	2.889672	3.39189	3.998125	2.514927	2.501657	2.552508	1.462466	1.713889	1.510458
GAPDH	2.348714	2.952631	1.630236	2.324188	2.377104	2.20029	2.294761	1.902645	2.444108

Figure 7B

	siNC			siACSL5-1			siACSL5-2		
ACSL5	0.95485	1.01312	1.03372	0.0292	0.0288	0.0314	0.01751	0.0176	0.01675

	siNC			siPPARG-1			siPPARG-2		
PPARG	0.97183	1.00815	1.02067	0.04865	0.04252	0.04607	0.04756	0.04143	0.04591

Figure 7L

	shSCR			shJAB1			shCUL4B		
CPT1A	0.98396	0.96371	1.05458	0.13998	0.14293	0.14096	0.09364	0.09967	0.10832
ACADM	0.99309	1.00696	1	0.26071	0.26619	0.26252	0.29741	0.30366	0.31004
ACOX1	0.96454	1.02264	0.94606	0.48185	0.47522	0.46544	0.25822	0.26548	0.26918
HADHA	1.04247	1.13288	1.08766	0.1776	0.17515	0.17155	0.11162	0.12046	0.12046
ACAA1	1.0163	1.10445	0.8909	0.13584	0.13213	0.13121	0.09672	0.10366	0.09672

s-Figure 7B

	siNC			siACSL5-1			siACSL5-2		
ACSL5	0.9982	1.00549	0.99634	0.22115	0.24633	0.28853	0.10691	0.08749	0.09805

	siNC			siPPARG-1			siPPARG-2		
PPARG	0.97292	1.01411	1.01354	0.25041	0.26369	0.24909	0.24865	0.24252	0.24607

s-Figure 7J

	shSCR			shJAB1			shCUL4B		
CPT1A	1	0.986233	1.013959	0.356109	0.3457	0.456584	0.397377	0.308508	0.325496
ACADM	1.042466	1.021012	0.939523	0.514152	0.485064	0.42445	0.33884	0.36588	0.313672
ACOX1	1.002313	1.002313	0.99539	0.482671	0.53025	0.530269	0.316463	0.228303	0.391525
HADHA	1.06437	0.97942	0.959264	0.379165	0.384584	0.373783	0.33884	0.33884	0.354364
ACAA1	0.990801	1.025741	0.983957	0.532884	0.543702	0.533033	0.470551	0.388843	0.440896
